# Human CSPG4-targeting CAR-macrophages inhibit melanoma growth

**DOI:** 10.1038/s41388-025-03332-0

**Published:** 2025-03-13

**Authors:** Daniel Greiner, Qian Xue, Trinity QA Waddell, Elena Kurudza, Piyush Chaudhary, Rachel L. Belote, Gianpietro Dotti, Robert L. Judson-Torres, Melissa Q. Reeves, Samuel H. Cheshier, Minna Roh-Johnson

**Affiliations:** 1 Department of Biochemistry, University of Utah School of Medicine, Salt Lake City, UT 84112 USA; 2 Department of Neurosurgery, Clinical Neurosciences Center, University of Utah, Salt Lake City, UT 84112 USA; 3 Huntsman Cancer Institute, University of Utah School of Medicine, Salt Lake City, UT 84112 USA; 4 Department of Pathology, University of Utah School of Medicine, Salt Lake City, UT 84112 USA; 5 Department of Molecular Genetics, The Ohio State University College of Arts and Sciences, Columbus, OH 43210 USA; 6 Department of Microbiology and Immunology, University of North Carolina, Chapel Hill, NC 27599 USA; 7 Department of Dermatology, University of Utah School of Medicine, Salt Lake City, UT 84112 USA; 8 Department of Oncological Sciences, University of Utah School of Medicine, Salt Lake City, UT 84112 USA; 9 Division of Pediatric Neurosurgery, Intermountain Primary Children’s Hospital, Salt Lake City, UT 84112 USA

**Keywords:** Melanoma, Innate immune cells

## Abstract

Approximately half of melanoma patients relapse or fail to respond to current standards of care, highlighting the need for new treatment options. Engineering T-cells with chimeric antigen receptors (CARs) has revolutionized the treatment of hematological malignancies but has been clinically less effective in solid tumors. We therefore sought to engineer alternative immune cell types to inhibit melanoma progression. Engineering macrophages with CARs has emerged as a promising approach to overcome some of the challenges faced by CAR-T cells; however, whether these engineered macrophages can effectively inhibit melanoma growth is unknown. To determine whether CAR-macrophages (CAR-Ms) specifically target and kill melanoma cells, we engineered CAR-Ms targeting chondroitin sulfate proteoglycan 4 (CSPG4), an antigen expressed in melanoma. CSPG4-targeting CAR-Ms exhibited specific phagocytosis of CSPG4-expressing melanoma cells. We developed 3D approaches to show that CSPG4-targeting CAR-Ms efficiently infiltrated melanoma spheroids. Furthermore, combining CSPG4-targeting CAR-Ms with strategies inhibiting CD47/SIRPα “don’t eat me” signaling synergistically enhanced CAR-M-mediated phagocytosis and robustly inhibited melanoma spheroid growth in 3D. Importantly, CSPG4-targeting CAR-Ms inhibited melanoma tumor growth in mouse models. These results suggest engineering macrophages against melanoma antigens is a promising solid tumor immunotherapy approach for treating melanoma.

## Introduction

Chimeric antigen receptor (CAR) T-cell therapy has demonstrated the groundbreaking potential to harness the immune system against cancer. While this revolutionary approach has reshaped the treatment of hematological malignancies, its promise has yet to be fully realized in solid tumors [1]. Several complexities specific to solid tumor biology present unique obstacles that have limited the success of CAR-T cell therapies. Tumor heterogeneity presents a formidable challenge for CAR-T cell therapy. Antigen heterogeneity in solid tumors means that CAR-T cells designed to target a single antigen may only eliminate cells with high antigen expression, leaving behind populations with low or no expression, resulting in therapeutic resistance and tumor relapse [2]. Solid tumors also have dense physical barriers of extracellular matrix that impede active T-cell infiltration [3] and cultivate a highly immunosuppressive microenvironment. This environment is characterized by the presence of suppressive immune cells (like regulatory T cells, myeloid-derived suppressor cells, and tumor-associated macrophages) [4, 5], inhibitory cytokines (such as TGF-beta) [6], hypoxia [7, 8], and metabolic factors that can disarm and exhaust CAR-T cells, ultimately limiting their anti-tumor activity [9]. While efforts to further engineer CAR-T cells for improved activity against solid tumors are ongoing, an alternative approach is engineering other immune cells, like macrophages, that inherently overcome these challenges.

Unlike T-cells, macrophages comprise a large portion of the overall mass in solid tumors [5]. Macrophages are professional phagocytes, eating their targets and easily infiltrating barriers surrounding solid tumors [10]. Macrophages can also reprogram other immune cells such as regulating the polarization of other macrophages [11] and educating adaptive immune cells through antigen presentation [10]. Given that macrophages phagocytose their tumor targets, the antigen presentation capabilities of macrophages suggest that macrophages will present multiple tumor antigens to T-cells, thus potentially bypassing challenges associated with tumor heterogeneity. The central role that macrophages play in the immune system suggests that macrophages are an ideal cell to use for adoptive cell therapies in cancer, as reprogramming the tumor immune response is likely to lead to a sustained and robust anti-tumor response.

Several groups have explored the potential of using chimeric antigen receptor-expressing macrophages (CAR-Ms) to treat various types of solid tumors. These CAR-Ms have predominantly targeted a variety of canonical tumor antigens including, but not limited to, CD19, CD47, HER2, and EGFRvIII [11–27]. These studies show that CAR-Ms targeting certain cancer types are specific and efficient, and CAR-Ms effectively reduce tumor burden in in vivo xenograft models. However, it is still unclear whether CAR-T-targeted antigens will universally apply to CAR-Ms, and whether all solid tumor types will be vulnerable to CAR-Ms. We opted to test the efficacy of CAR-Ms in the context of melanoma, given the high degree of antigen heterogeneity observed in melanoma [28–30]. Thus, we sought to engineer CAR-Ms against a melanoma-specific antigen first to determine whether CAR-Ms could target melanoma and, second, whether CAR-Ms inhibit melanoma growth.

Numerous tumor-associated antigens enriched in melanomas have been identified, and we focused on chondroitin sulfate proteoglycan 4 (CSPG4), also known as NG2 and HMW-MAA [31]. CSPG4 is a ~ 300 kDa transmembrane proteoglycan that regulates cancer cell migration, invasion, epithelial-mesenchymal transition, and proliferation [32]. CSPG4 is frequently highly expressed in melanoma tumors, and expression in non-malignant cells is low, making it an ideal target antigen [32, 33]. Furthermore, a CAR-T cell therapy targeting CSPG4 is currently used in a phase I clinical trial for head and neck cancer (NCT06096038) [34], suggesting CSPG4-targeting CARs are specific with minimal off-target effects. Thus, we selected CSPG4 as a target for proof-of-principle approaches to determine whether melanoma-targeting CAR-Ms will phagocytose melanoma cells and inhibit melanoma growth.

In this study, we engineered CSPG4-targeting CAR-Ms from human blood donors. We demonstrate that CSPG4-targeting CAR-Ms phagocytose melanoma cells in vitro, and that this CAR-M-mediated phagocytosis is specific to CSPG4-expressing cells. We developed 3D approaches and show that CSPG4-targeting CAR-Ms in combination with CD47 blocking antibodies efficiently inhibit melanoma spheroid growth in 3D. Furthermore, we show that CSPG4-targeting CAR-Ms inhibit melanoma growth in vivo. Thus, this work highlights the potential of CAR-Ms as a therapy for melanoma patients.

## Results

### CSPG4-targeting CAR-Ms phagocytose CSPG4-expressing cancer cells

**Fig. 1:**
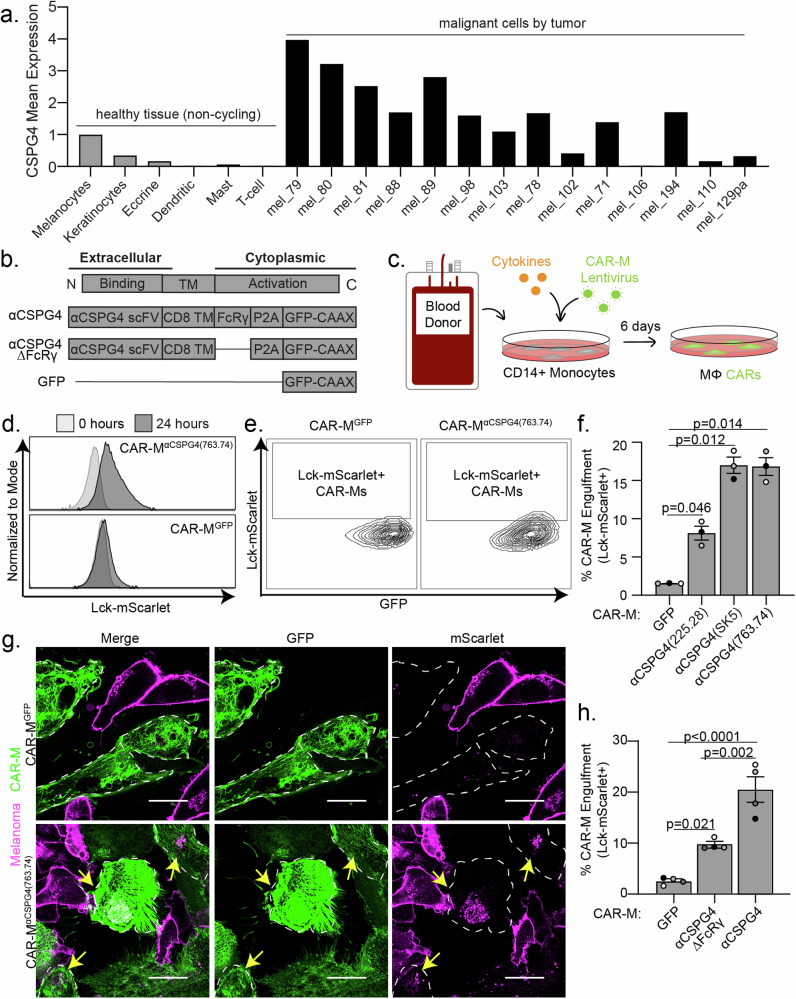
CSPG4-targeting CAR-Ms engulf CSPG4-exressing melanoma fragments in 2D cell culture. **a** CSPG4 transcript levels by single-cell RNA sequencing analysis of healthy (non-cycling) tissue vs melanoma tumors, rank mean normalized. **b** Schematic of chimeric antigen receptor macrophage constructs. **c** Schematic for generation of primary human CAR-Ms. **d** Representative histograms showing phagocytic events after 24 h coculture with CAR-M^GFP^ or CSPG4^αCSPG4(763.74)^. **e** Representative flow cytometry contour plots showing CAR-M-mediated engulfment of Lck-mScarlet fragments by CAR-M^GFP^ control and CSPG4^αCSPG4(763.74)^. **f** Quantification of the % CAR-Ms that engulfed melanoma Lck-mScarlet fragments (% CAR-M Engulfment) shown in (**d**, **e**) for CAR-M^GFP^ and CAR-M with 3 CSPG4-targeting scFvs (225.28, SK5, 763.74). **g** Representative single z-plane images of CAR-M^αCSPG4(763.74)^ (green)-mediated engulfment of A375-Lck-mScarlet cells (magenta) after 24 h of 2D coculture by microscopy. White dashed line outlines CAR-M^αCSPG4^ cell boundary, and yellow arrows indicate CAR-M eating events. Scale bar is 20 microns. **h** Quantification of CSPG4^αCSPG4(763.74)^ Lck-mScarlet engulfment events compared to CAR-M^GFP^ and CAR-M^αCSPG4(763.74)ΔFcRγ^ by flow cytometry. For all graphs, each dot on the graph is an independent PBMC donor (biological replicate), each as a shade of gray. **f**, **h** Mean +/− SEM, 1-way ANOVA with Tukey’s multiple comparisons test.

CSPG4 was first characterized as overexpressed in melanoma; however, CSPG4 upregulation in numerous other cancers, including breast and glioblastoma, has since been described [31, 35]. CSPG4 has been reported to promote tumor growth and survival through numerous signaling pathways associated with its extracellular domains [35]. Transcriptional analysis suggests that CSPG4 mRNA expression is low in healthy tissue, with undetectable protein expression levels by immunohistochemistry [33]. We analyzed published single-cell RNA-sequencing data from fresh healthy human skin and metastatic melanomas [36–38]. These analyses revealed significantly higher CSPG4 transcript abundance in a majority of malignant tumor cells from patient samples compared to healthy skin cells, including melanocytes (Fig. 1a; Supplemental Fig. 1a). Importantly, non-malignant cells, both in the tumor microenvironment (Supplemental Fig. 1b) and across broader cell types (Supplemental Fig. 1c), generally presented low CSPG4 transcript counts, with notable exceptions being Sertoli cells, oligodendrocyte precursors cells, and a population of smooth muscle cells. However, immunohistochemistry analyses in oligodendrocyte precursor cells and smooth muscle cells revealed that CSPG4 protein expression is significantly lower in these tissues versus cancer cells [39–42], and the blood-testis barrier represents one of the tightest blood-tissue barriers in the body, protecting Sertoli cells [43]. These results suggest that CSPG4-targeting CAR-Ms will not result in off-target effects resulting in adverse consequences. In support of this hypothesis, rats treated with antibodies against CSPG4 did not exhibit long-term toxicity, and CSPG4 knockout mice are viable and exhibit no gross deformities [44, 45]. These findings have prompted the initiation of a phase I clinical trial using CSPG4-targeting CAR-T cells in head and neck squamous cell carcinoma [34]. Thus, given the high expression of CSPG4 on melanoma cells, we sought to use this established tumor antigen to test whether engineering macrophages to target an antigen on melanoma cells will lead to high macrophage infiltration into melanoma tumors and robust melanoma phagocytosis and clearance.

To target CSPG4-expressing cells, we engineered CAR constructs incorporating one of three distinct single-chain variable fragments (scFv) exhibiting affinity for CSPG4: 225.28, SK5, and 763.74, each of which recognizes different extracellular domains [33, 46–48] (Supplemental Fig. 2a). We followed previous CAR designs [12] using an FcRγ signaling domain and combined this domain with CSPG4 scFvs. As controls, we designed CARs lacking the intracellular FcRγ phagocytic signaling domain, as well as a CAR expressing GFP alone, to control for activation associated with transduction (Fig. 1b). To generate CAR-Ms, we used human primary blood monocyte-derived macrophages, which we isolated from healthy blood donors, transduced monocytes with CSPG4-targeting CARs, and then differentiated the monocytes into macrophages (Fig. 1c). Using our previously established protocols [49], CSPG4-CAR expressing macrophages were generated with high transduction efficiencies (Supplemental Fig. 2b), and thus we proceeded with using primary macrophage-derived CAR-Ms for all subsequent studies.

We used the human metastatic melanoma cell line A375 to test whether CSPG4-targeting CAR-Ms phagocytose melanoma cells. A375 cells exhibit high CSPG4 expression compared with primary macrophages from multiple donors as a negative control (Supplemental Fig. 2c). We generated A375 expressing a fluorescent membrane marker (Lck-mScarlet) to visualize the melanoma cells. We then cocultured A375-Lck-mScarlet cells with each of the three different CSPG4-targeting CAR-Ms. Using flow cytometry to quantify CAR-M-mediated eating as the percentage of CAR-Ms with A375-Lck-mScarlet fragments (Supplemental Fig. 2d), we found that each CAR-M^αCSPG4(225.28/SK5/763.74)^ engulfed Lck-mScarlet melanoma fragments at higher rates than the control CAR-M^GFP^ (Fig. 1d–f). These differences in engulfment rates were not due to the level of CAR transduction efficiency, as the percent CAR transduction efficiency in macrophages did not affect the percent of CAR-M-mediated engulfment (Supplemental Fig. 2e). However, the level of CAR expression affected the ability of CAR-Ms to engulf melanoma cells. CAR-Ms expressing higher levels of the CSPG4-targeting CAR exhibited higher rates of melanoma engulfment compared to CAR-Ms expressing lower levels of the CSPG4-targeting CAR (Supplemental Fig. 2f, g).

To confirm that flow cytometric events are bona fide CAR-M-mediated engulfment events, rather than A375 membrane fragments adhering to the exterior of CAR-Ms, we imaged CAR-M and A375 cocultures with high-resolution microscopy. After 24 h of coculture, we observed Lck-mScarlet punctae inside of CAR-M^αCSPG4(225.28/SK5/763.74)^ cells compared to CAR-M^GFP^ (Fig. 1g; Supplemental Fig. 2h). We proceeded to use CAR-M^αCSPG4(763.74)^ (herein referred to as CAR-M^αCSPG4^) for the remainder of our studies due to the consistency of target cell engulfment, availability of published data regarding its binding region on CSPG4 [50], and its current use in phase I CAR-T cell clinical trials (NCT06096038) [34]. To better understand CAR-M-mediated engulfment of melanoma fragments, we used timelapse recordings and 3D visualization approaches. Using 3D reconstruction, we confirmed that the Lck-mScarlet punctae were fully internalized in the CAR-M^αCSPG4^ (Supplemental Fig. 3a; Supplemental Video 1). Live timelapse recordings reveal that CAR-M^αCSPG4^ began engulfing melanoma fragments within the first 4 h of coculture, with most events being trogocytosis (cell nibbling), rather than whole-cell phagocytosis (Supplemental Fig. 3b; Supplemental Video 2).

We next tested whether the intracellular FcRγ region was critical for CAR-M-mediated engulfment. Consistent with previous studies with CD19-targeting CAR-Ms [12], removal of the FcRγ domain (αCSGP4ΔFcRγ) resulted in a statistically significant reduction in the percent engulfment by flow cytometry compared to the full CSPG4-targeting CAR-M (Fig. 1h). Interestingly, however, CAR-M^αCSGP4ΔFcRγ^ exhibited higher rates of engulfment than control CAR-M^GFP^ (Fig. 1h). We hypothesize that the physical interaction between the CSPG4-scFv and CSPG4 on the surface of melanoma cells is sufficient to promote CAR-M-mediated engulfment of melanoma fragments, likely via the maintenance of endogenous FcRγ signaling. Live cell imaging of CAR-M^αCSGP4ΔFcRγ^ cocultures with A375-Lck-mScarlet cells also show higher engulfment rates than CAR-M^GFP^ (Supplemental Fig. 3c, d). Taken together, these results suggest that CSPG4-targeting CAR-Ms efficiently engulf metastatic melanoma cell fragments.

**Fig. 2:**
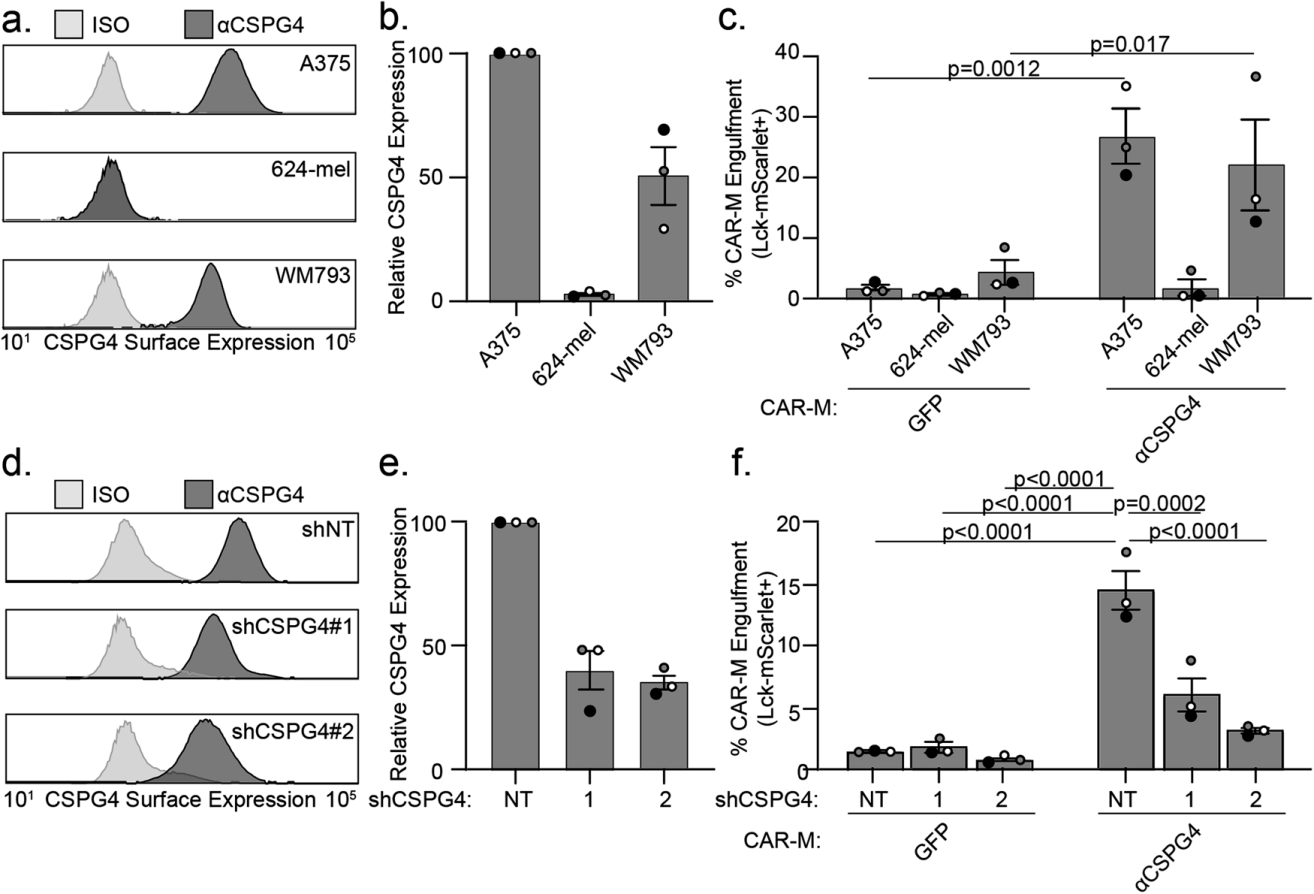
CSPG4-targeting CAR-M engulfment of melanoma fragments is specific to CSPG4-expressing melanoma cells. **a** Representative flow plot of CSPG4 surface expression on A375, 624-mel, and WM793 cells with flow cytometry. **b** Quantification of CSPG4 surface expression in (**a**) normalized to A375 expression. **c** Quantification of CAR-M-mediated engulfment events (CAR-M^GFP^ and CAR-M^αCSPG4^) of A375, 624-mel, and WM793 cells after 24 h of coculture. **d** A representative flow plot of CSPG4 surface expression on A375 cells expressing non-targeted shRNA or 2 different shCSPG4 RNAs compared to isotype controls (ISO). **e** Quantification of CSPG4 surface expression in (**d**) normalized to shNT expression. **f** Quantification of CAR-M-mediated engulfment events (CAR-M^GFP^ and CAR-M^αCSPG4^) of A375 cells treated with non-targeting shRNAs or 2 different shCSPG4 RNAs. For all graphs, each dot on the graph is an independent PBMC donor (biological replicate), each as a shade of gray. **c** Mean +/− SEM, 2-way ANOVA with Sidak’s multiple comparisons test. **f** Mean +/− SEM, 2-way ANOVA with Tukey’s multiple comparisons test. Non-significant comparisons are not indicated on the graph.

We next sought to determine the specificity of CSPG4-CAR-M-mediated engulfment. Using human melanoma cell lines with differing CSPG4 surface expression, we quantified the level of cancer cell engulfment by CAR-M^αCSGP4^ and CAR-M^GFP^. We found that A375 cells and WM793 melanoma cells exhibited high levels of CSPG4 expression on the surface of the cells, and 624-mel melanoma cells exhibited low CSPG4 expression (Fig. 2a, b). When we cocultured each cell line with control CAR-M^GFP^, we observed no differences in CAR-M-mediated engulfment. However, when we cocultured each of these cell lines with CAR-M^αCSPG4^, we observed high rates of engulfment with A375 and WM793 cells, but not with 624-mel cells (Fig. 2c). To further confirm specificity, we knocked down CSPG4 expression in A375 cells. We knocked down A375 surface expression with two different CSPG4 shRNAs to ~40% compared to non-targeting controls (Fig. 2d, e). We found that the percent of CAR-M^αCSPG4^ cells engulfing Lck-mScarlet fragments was significantly reduced when CSPG4 surface expression was decreased on A375 cells compared to non-targeting controls (Fig. 2f). These results suggest that CAR-M^αCSPG4^-mediated engulfment is specific to CSPG4-expressing cells.

### CSPG4-targeting CAR-Ms alone do not cause reduced melanoma cell survival

**Fig. 3:**
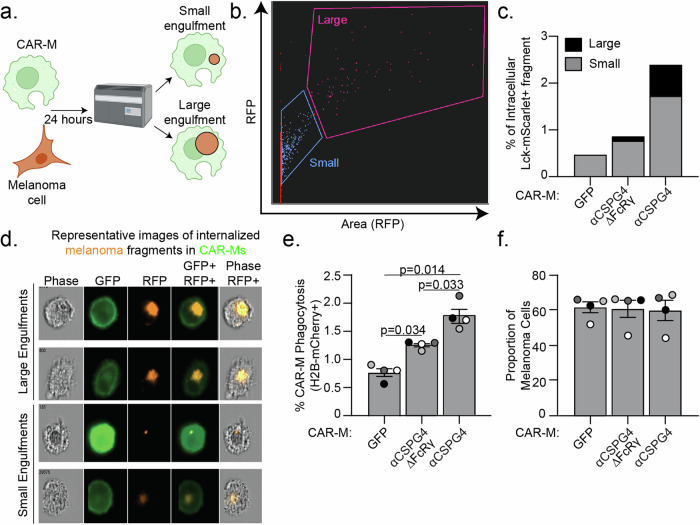
CSPG4-targeting CAR-Ms primarily trogocytose target melanoma cells. **a** Schematic (created with Biorender.com) of CAR-M coculture with melanoma cells and analysis of engulfment size by image-based flow cytometry (Imagestream, Cytek Amnis). **b** Representative gating of the size of internalized A375-Lck-mScarlet fragments in CAR-Ms, images acquired with a 63X objective lens on the Imagestream. **c** Quantification of data in (**b**) – small ( < 75 μm^2^) versus large ( > 75 μm^2^) A375-Lck-mScarlet fragments inside CAR-Ms. *n* = 1 PBMC donor. **d** Representative images of “small” and “large” A375-Lck-mScarlet fragments (red) inside CAR-Ms (green). **e** Quantification of p**e**rcent CAR-M whole-cell phagocytosis of A375-H2B-mCherry cells. **f** Quantification of proportion of live, A375-H2B-mCherry cells remaining in the coculture population by flow cytometry after 24 hours of coculture with CAR-M^αCSPG4^ compared to CAR-M^GFP^. Each dot on the graph is an independent PBMC donor (biological replicate), each as a shade of gray. **e**, **f** Mean +/− SEM, 1-way ANOVA with Tukey’s multiple comparison test. Non-significant comparisons are not indicated on the graph.

We next determined whether CAR-M^αCSPG4^ engulfment affected melanoma cell survival. Since nibbling/trogocytosis of target cells often does not result in cell death, we first quantified the amount of melanoma cell trogocytosis, and compared this rate to that of whole-cell phagocytosis. The median area of Lck-mScarlet melanoma cell fragments internalized in CAR-M^αCSPG4^ cells is significantly lower (65 μm^2^) than the median area of a whole, unengulfed Lck-mScarlet melanoma cells (473 μm^2^) (Supplemental Fig. 3e), suggesting that we were predominantly imaging trogocytosis events and not whole-cell phagocytosis events. We next took advantage of image-based flow cytometry, where we could both mask for complete internalization, and quantify the relative size of the internalized fragments across thousands of cells (Fig. 3a, b). Based on the engulfment size measurements (Supplemental Fig. 3e), we stratified engulfment events as CAR-Ms with large melanoma fragments ( > 75 μm^2^) versus CAR-Ms with small melanoma fragments ( < 75 μm^2^). We observed both a higher percent of overall Lck-mScarlet engulfment, as well as a higher proportion of CAR-Ms with large melanoma fragments in CAR-M^αCSPG4^ compared to control CAR-M^GFP^ (Fig. 3c, d; Supplemental Fig. 4). To specifically quantify whole-cell phagocytosis, we generated A375 cells expressing a fluorescently-tagged histone H2B in the nucleus (H2B-mCherry), allowing us to measure nuclear engulfment as a read-out for whole-cell phagocytosis. Consistent with the image-based flow cytometry observations, this analysis revealed increased levels of whole-cell phagocytosis by CAR-M^αCSPG4^ versus CAR-M^αCSGP4ΔFcRγ^ or CAR-M^GFP^ controls (Fig. 3e). However, the overall level of whole-cell phagocytosis was low, at less than 2%. We then analyzed melanoma cell survival as the proportion of melanoma cells remaining in the coculture population and observed no significant changes in the overall percentage of melanoma cells in the coculture population across all conditions (Fig. 3f). These results suggest that although CAR-M^αCSPG4^ exhibited higher rates of whole-cell melanoma phagocytosis compared to control CAR-Ms, these phagocytic events were not sufficient to reduce melanoma cell survival.

### Combining CSPG4-targeting CAR-Ms with αCD47 inhibits melanoma growth in 3D

**Fig. 4:**
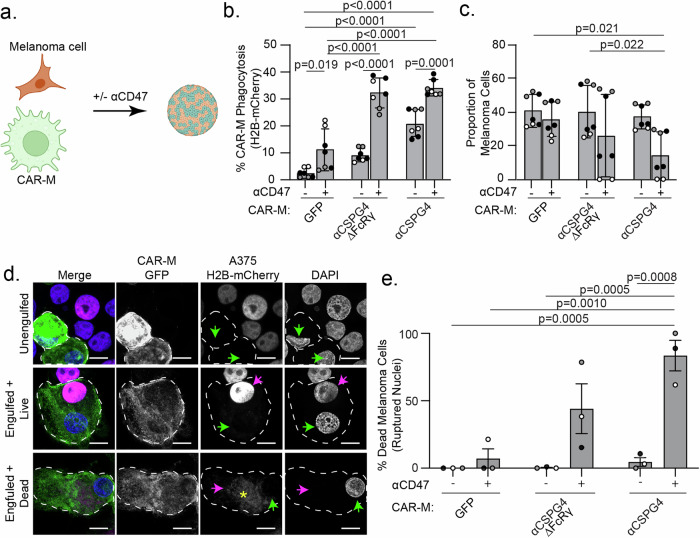
Combining CSPG4-targeting CAR-Ms with αCD47 leads to melanoma cell death in 3D. **a** Schematic (created with Biorender.com) of CAR-M and melanoma cells co-forming 3D spheroids with αCD47 (+) or with IgG isotype control antibodies (−). **b** Quantification of percent CAR-M whole-cell phagocytosis of melanoma cells in spheroids after 72 h. **c** Proportion of melanoma cells remaining in spheroids after 72 h coculture by flow cytometry. **d** Representative images of replated spheroids showing “unengulfed” melanoma cells (single spherical nuclei, colocalization with H2B-mCherry, no engulfment by CAR-M); “engulfed + live” (melanoma engulfment by CAR-M, A375-H2B-mCherry nuclei intact and colocalizing with DAPI); or “engulfed + dead” (melanoma engulfment by CAR-M, A375-H2B-mCherry does not colocalize with DAPI). White dashed outline marks individual CAR-Ms. Green arrows indicate CAR-M nuclei. Magenta arrows indicate the A375-H2B-mCherry signal colocalizing with DAPI inside CAR-Ms. The yellow asterisk indicates dispersed A375-H2B-mCherry signal not colocalizing with DAPI inside CAR-Ms. Scale bar indicates 10 microns. **e** Quantification of ruptured nuclei as percent of total melanoma cells after 72 h of coculture. CAR-M^GFP^/IgG (*N* = 104), CAR-M^GFP^/αCD47 (*N* = 70), CAR-M^αCSPG4ΔFcRγ^/IgG (*N* = 110), CAR-M^αCSPG4ΔFcRγ^/αCD47 (*N* = 47), CAR-M^αCSPG4^/IgG (*N* = 65), CAR-M^αCSPG4^/ αCD47 (*N* = 34). **b**, **c**, **e** *N* = 3 independent PMBC donors (biological replicates); each shade of gray indicates one biological replicate, with each dot indicating technical replicates. Mean +/− SEM, 2-way ANOVA with Tukey’s multiple comparisons test. Non-significant comparisons are not indicated on the graphs.

Puzzled by the lack of change in melanoma cell survival in our system, we next sought to test the effects of CAR-Ms in a more physiologically relevant 3D environment. Macrophages in suspension with low membrane tension exhibit a much greater capacity for phagocytosis compared to macrophages adhered and spread on tissue culture dishes [51]. Additionally, many cancer cells, including melanoma, overexpress CD47 on their surface, a “don’t eat me” signal [52]. Melanoma-expressed CD47 interacts with SIRPα on macrophages, protecting the melanoma cells from phagocytosis [52]. Blocking CD47/SIRPα signaling has been shown to inhibit A375 tumor growth in mice [53]. Therefore, we hypothesized that elevated expression of CD47 in A375 cells inhibits CAR-M^αCSPG4^-mediated whole-cell phagocytosis, so we sought to inhibit CD47 with a commercially available humanized IgG4 monoclonal antibody that was being used in clinical trials (αCD47) [54–56]. We first cultured CAR-M/melanoma spheroids (Fig. 4a) using A375 cells expressing Lck-mScarlet and quantified CAR-M-mediated engulfment with image-based flow cytometry after 24 hours. In 3D cultures with CAR-M^αCSPG4^, we observed almost 50% of CAR-Ms were positive for Lck-mScarlet fragments at 24 hours, with a higher proportion of CAR-Ms with large, internalized melanoma fragments when the CD47/SIRPα axis was inhibited with αCD47 compared to those treated with IgG isotype control antibodies (Supplemental Fig. 5a). Control CAR-M^GFP^ did not show altered phagocytosis of melanoma cells in the presence of αCD47 (Supplemental Fig. 5a). These results suggest that in 3D cultures, CAR-M^αCSPG4^ and αCD47 work cooperatively to enable phagocytosis. To test between trogocytosis and whole-cell phagocytosis, we again used melanoma cells with fluorescently tagged nuclei (H2B-mCherry). We observed a significant increase in the percent of CAR-M-mediated whole-cell phagocytosis by CAR-M^αCSPG4^ compared to control CAR-M^GFP^, and that this whole-cell phagocytosis was further increased to ~35% in the presence of αCD47 versus isotype controls (Fig. 4b). Excitingly, we found that the proportion of melanoma cells in the final spheroid was significantly reduced in the presence of CAR-M^αCSPG4^ with αCD47 (Fig. 4c). To determine whether the lack of melanoma cells was due to CAR-M-mediated melanoma cell death, we performed the 3D spheroid experiments, and instead of processing the cells for flow cytometry, we dissociated the spheroids to visualize the cells with high-resolution microscopy. Dead/dying cells exhibit ruptured and fragmented nuclei, and histones become separated from DNA [57]. Thus, we quantified the number of internalized melanoma cells with ruptured H2B-mCherry nuclei as a measure of cell death. We observed a population of A375-H2B-mCherry cells that were not phagocytosed by CAR-Ms (“unengulfed”); a population of A375-H2B-mCherry cells that were engulfed by CAR-Ms, but the nuclei remained intact with the H2B-mCherry signal colocalizing with DAPI (“engulfed+live”); and a population of A375-H2B-mCherry cells that were engulfed by CAR-Ms with their nuclei broken down and H2B-mCherry no longer localized with DNA, indicating cell death (“engulfed+dead”). We found that a significantly higher proportion of A375 cells exhibited the “engulfed+dead” phenotype in cultures with CAR-M^αCSPG4^ and αCD47, compared to all other conditions (Fig. 4d, e; Supplemental Fig. 5b-d). Interestingly, the reduction in melanoma cells and robust CAR-M whole-cell phagocytosis with αCD47 was only observed in 3D culture conditions and not in 2D cultures (Supplemental Fig. 5e-i). These results suggest that the combination of CAR-M^αCSPG4^ with αCD47 in 3D leads to high levels of melanoma cell death.

**Fig. 5:**
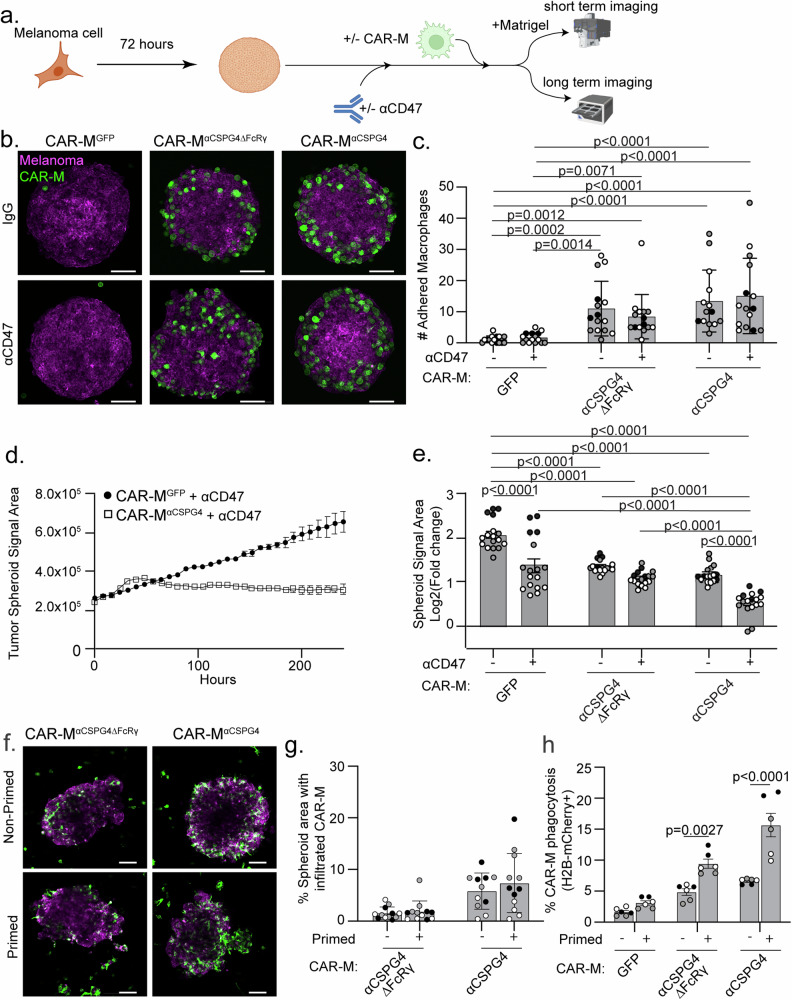
Combining CSPG4-targeting CAR-Ms with αCD47 inhibits melanoma spheroid growth in 3D. **a** Schematic (created with Biorender.com) of a pre-formed A375 melanoma spheroid with CAR-Ms in combination with αCD47 (+) or isotype control antibodies (−) for short-term adherence assays and long-term imaging. **b** Representative images of pre-formed A375-Lck-mScarlet spheroids (magenta) cultured with CAR-Ms (green) (following schematic in (**a**)) for 4–8 h with αCD47 (+) or IgG isotype controls (−) and embedded in Matrigel for imaging. Scale bar is 100 microns. **c** Quantification of GFP + CAR-Ms adhered to mScarlet+ melanoma spheroids in (**b**). **d** Representative plot of A375-H2B-mCherry spheroid growth in culture with CSPG4-targeting CAR-Ms or control CAR-Ms with αCD47 over 10 days, as measured by mCherry area over time. **e** Quantification of data in (**d**) at day 10 across conditions (N = 17 spheroids across N = 3 independent PBMC donors (biological replicates)). Data is shown as log_2_(fold change from time zero). **f**, **g** Representative images (**f**) and quantification (**g**) of infiltration of primed vs non-primed CAR-Ms (CAR-M^GFP^ or CAR-M^αCSPG4^, green) into A375-Lck-mScarlet spheroids (magenta). **h** Quantification of CAR-M-mediated engulfment events by primed or non-primed CAR-Ms (CAR-M^GFP^ and CAR-M^αCSPG4^) of A375 cells in a 3D spheroid at 72 h. For graphs, each shade of gray indicates an independent PBMC donor (biological replicate), with each dot indicating technical replicates. **c** Mean +/− SEM, Kruskal-Wallis with Dunn’s multiple comparisons test. Non-significant comparisons are not indicated on the graph. **e** Mean +/− SEM, 2-way ANOVA with Tukey’s multiple comparisons test. Non-significant comparisons are not indicated on the graph. **g**, **h** Mean +/− SEM, 2-way ANOVA with Sidak’s multiple comparisons test. Non-significant comparisons are not indicated on the graph.

To better mimic tumor growth conditions, we altered the 3D approaches and allowed melanoma spheroids to form prior to the addition of CAR-Ms (Fig. 5a). After 4–8 h of CAR-M addition, we removed the spheroids from the U-bottom low-attachment plates and embedded them in Matrigel to visualize CAR-M interactions with the melanoma spheroid. We found that CAR-M^αCSPG4^ remain adhered to the melanoma spheroids when spheroids were transferred to Matrigel, whereas the CAR-M^GFP^ did not (Fig. 5b, c). Adding αCD47 to the culture did not affect CAR-M attachment to the spheroid. Using high resolution imaging, we further observed that CAR-M^αCSPG4^ infiltrate the spheroid (Supplemental Fig. 6a; Supplemental Video 3) and phagocytose melanoma cells within the spheroid (Supplemental Fig. 6b).

We next tested how CAR-Ms affect melanoma spheroid growth over time. We grew melanoma spheroids for 72 h, added CAR-Ms, and then allowed spheroids to continue to grow for 10 days in the U-well low-attachment plates (Fig. 5a). We found that melanoma spheroid growth was inhibited when treated with CAR-M^αCSPG4^ compared to control CAR-M^GFP^ (Fig. 5e, comparing bar 1 & bar 5). This spheroid inhibition was not limited to melanoma, as CSPG4-positive breast cancer spheroids also exhibited reduced spheroid growth in the presence of CAR-M^αCSPG4^ compared to control CAR-M^GFP^ (Supplemental Fig. 7a, b). As expected, the growth of CSPG4-negative breast cancer spheroids was unaffected by CAR-M^αCSPG4^ (Supplemental Fig. 7c, d), showing that CSPG4-targeting CAR-Ms specifically regulated the growth of CSPG4-expressing cancer cells.

We then asked whether spheroid growth was affected when combining CAR-M^αCSPG4^ with αCD47. We observed a synergistic effect in which combining CAR-M^αCSPG4^ with αCD47 resulted in robust inhibition of melanoma spheroid growth (Fig. 5d, e; Supplemental Fig. 8a, b). Notably, CAR-M^αCSPG4^ infiltrated the melanoma spheroid; whereas, CAR-M^GFP^ localized primarily to the exterior of the melanoma spheroid (Supplemental Fig. 8c, d; Supplemental video 4), suggesting increased interactions between spheroids and CSPG4-targeting CAR-Ms versus control CAR-Ms. We next sought to determine whether this inhibition of spheroid growth was specific for cells expressing CSPG4 by knocking down CSPG4 expression. However, consistent with published literature, the A375 cells expressing the shCSPG4 clones failed to form viable spheroids and disassembled during media changes, thus precluding analysis (Supplemental Fig. 8e, f) [58]. Taken together, these results suggest that combining CSPG4-targeting CAR-Ms with αCD47 approaches result in robust inhibition of melanoma growth in 3D by increased phagocytosis.

The dramatic synergistic effects of combining CSPG4-targeting CAR-Ms with αCD47 led us to question whether we could reduce the concentration of αCD47, and still observe a robust CAR-M-mediated whole-cell phagocytosis. Reducing the αCD47 concentration, while still showing efficient CAR-M-mediated phagocytosis of cancer cells, may reduce off-target effects when used in patients, given that almost all healthy cells express some level of CD47. We started with 10 µg/mL of αCD47 as used in previous work [56, 59] and performed a serial dilution series. Combining αCD47 with control CAR-M^GFP^ showed no significant increase in melanoma whole-cell phagocytosis until 10 µg/mL of αCD47 (Supplemental Fig. 8g; white bar at 10^4^ compared to IgG control). At this αCD47 concentration, we also observed a significant increase in CAR-M^αCSPG4^-mediated whole-cell phagocytosis compared to CAR-M^GFP^ control (Supplemental Fig. 8g; black bar vs white bar at 10^4^). This level of CAR-M^αCSPG4^-mediated phagocytosis did not decrease until the concentration of αCD47 was reduced to 100 ng/mL αCD47 (Supplemental Fig. 8g; comparing black bars at 10^4^ to 10^2^). These results suggest that significantly decreased concentrations of αCD47 still enhance CSPG4-CAR-M-mediated whole-cell phagocytosis of melanoma cells, but not with CAR-M^GFP^.

We next asked whether culturing CAR-Ms with cancer cells “primes” CAR-Ms for enhanced cancer cell killing given work demonstrating Fc receptor priming [27]. We cocultured CSPG4-targeting CAR-Ms with cancer cells for 48 h, FACS-isolated these CAR-Ms (“primed”), and evaluated infiltrative ability and phagocytosis capacity compared to CAR-Ms that had not interacted with cancer cells (“non-primed”). We again first grew A375-H2B-mCherry spheroids for 72 h, and then added “primed” or “non-primed” CAR-Ms. When we quantified CAR-M infiltrative abilities by quantifying the area of CAR-Ms that successfully infiltrated melanoma spheroids, we found no difference between “primed” CAR-Ms and “non-primed” CAR-Ms (Fig. 5f, g). However, “primed” CAR-Ms exhibited significantly higher rates of melanoma phagocytosis compared to “non-primed” CAR-Ms, with the highest rates of phagocytosis observed with “primed” CAR-M^αCSPG4^ (Fig. 5h). Together, these results suggest that CAR-Ms that have previously interacted with cancer cells subsequently display increased phagocytic ability.

### CSPG4-targeting CAR-Ms inhibit melanoma growth in vivo

Given our in vitro findings, we sought to use a syngeneic system using murine melanoma cells and murine bone marrow-derived macrophages. We measured the expression of CSPG4 in several mouse melanoma cell lines by flow cytometry and observed high CSPG4 expression in the YUMM1.7 and YUMM1.1 cell lines (Supplemental Fig. 9a) [60]. We therefore generated YUMM1.7 cells expressing H2B-mCherry. We then determined which CSPG4 scFv to use for these murine studies. The 763.74 CSPG4 scFv has no demonstrated reactivity with the murine CSPG4 homolog, likely due to a two amino acid difference between human and mouse CSPG4 in the binding epitope (Supplemental Fig. 9b). Conversely, the 225.28 binding epitope only has a one amino acid difference between human and mouse CSPG4 and has previously demonstrated some cross-species reactivity [44]. Thus, we first tested whether CAR-Ms generated with the 225.28 scFv phagocytosed murine YUMM1.7-H2B-mCherry melanoma cells in 3D (Supplemental Fig. 9c). CAR-M^αCSPG4(225.28)^ exhibited a low rate of phagocytosis, with only ~4% of CAR-M^αCSPG4(225.28)^ phagocytosing YUMM1.7-H2B-mCherry cells (Supplemental Fig. 9c), a rate similarly observed in CAR-M^αCSPG4(763.74)^ and control CAR-M^GFP^ cells. These results suggest that CAR-M^αCSPG4(225.28)^ do not effectively target murine CSPG4, and thus precluded our ability to test the function of CAR-M^αCSPG4(225.28)^ in syngeneic mouse models. We therefore turned our attention to testing human CAR-Ms and human melanoma cells in immune-compromised models.

**Fig. 6:**
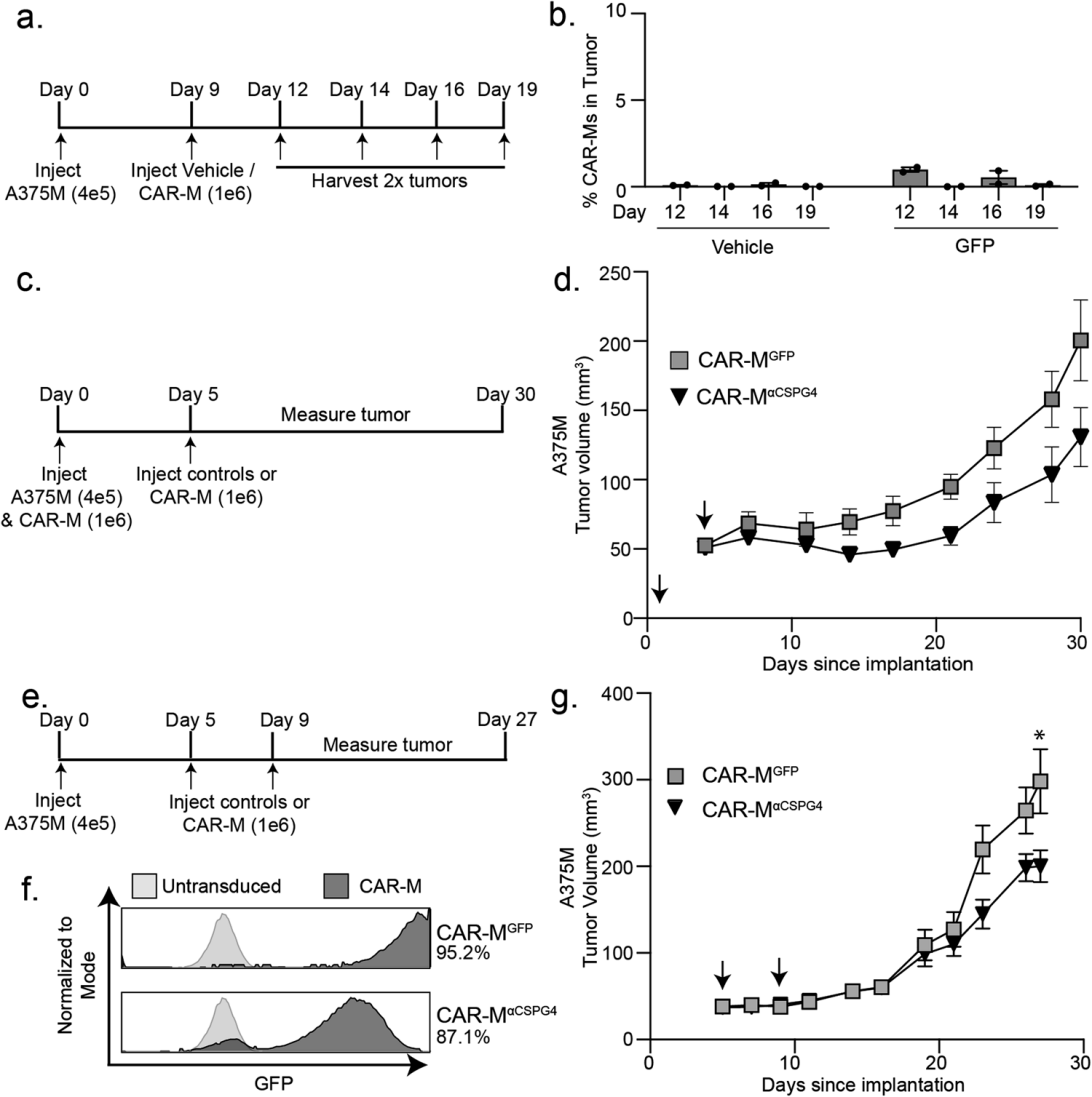
αCSPG4 CAR-Ms inhibit melanoma growth in vivo. **a** Experimental outline showing that A375-H2B-mCherry cells were injected in NRG mice, and then injected with CAR-M^GFP^ after 9 days. Tumors were dissociated and processed for flow cytometry on indicated days to determine presence of CAR-Ms. **b** Quantification of the presence of CAR-M^GFP^ in tumors over time in (**a**). **c** Experimental outline showing A375-H2B-mCherry same-day injections with CAR-M^αCSPG4^ or CAR-M^GFP^ in NRG mice (*N* = 4 mice per group). **d** Tumor volume for (**c**) plotted over time. Arrows indicate CAR-M injections. **e** Experimental outline showing CAR-M^αCSPG4^ or CAR-M^GFP^ injections after A375-H2B-mCherry engraftment in NRG mice (*N* = 6 mice per group). **f** Flow cytometry plots showing percent human macrophage transduction for CAR-M^GFP^ and CAR-M^αCSPG4^. **g** Tumor volume for (**e**, **f**) plotted over time. Arrows indicate CAR-M injections. **d**, **g** 1-way ANOVA with Tukey’s multiple comparisons test at terminal time point. *p < 0.05 versus indicated group.

We determined whether CAR-M^αCSPG4(763.74)^ inhibits A375 human melanoma growth in immune-compromised NRG mice [61]. We opted to inject CAR-Ms peri-tumorally, rather than systemically [11], to maximize on-target effects, while minimizing potential off-target effects associated with intravenous administration. We first determined how long CAR-Ms are detectable in the tumor to determine the treatment regimen. We reasoned that the CAR-Ms did not need to stay in the tumor for the entirety of the experiment, but CAR-Ms must be present in the tumor long enough to phagocytose CSPG4-positive melanoma cells and potentially reprogram the tumor environment for a sustained response. Thus, we injected 400 K A375 cells into the right flank of 6-8 week-old female NRG mice, waited for tumors to become palpable, and then peri-tumorally injected 1 M CAR-Ms on day 9. We then euthanized mice on days 12, 14, 16, and 19 to determine whether CAR-Ms were present in the tumor by flow cytometry (Fig. 6a). We found that CAR-Ms were present at 1% of the total live cells in the tumor after 3 days post-injection (day 12), and that this number dropped to almost zero starting at 5 days post-injection (day 14) (Fig. 6b). Thus, we proceeded with a two-stage CAR-M injection strategy, 3–5 days apart.

We first tested subcutaneous same-day injections of A375-H2B-mCherry cells and CAR-Ms into the flanks of mice, as same-day injection approaches were routinely used in previously published CAR-M work [11, 13, 14, 17]. We injected 400 K A375-H2B-mCherry cells and 1 M CAR-M^αCSPG4^, CAR-M^GFP^, or vehicle control and then re-injected with only CAR-Ms or vehicle control 5 days later (Fig. 6c). We found no differences in animal weight across conditions, suggesting no gross off-target toxicity (Supplemental Fig. 10a). We found that treating mice with CAR-M^αCSPG4^ inhibited the growth of the tumor compared to CAR-M^GFP^ controls, although these differences were not statistically significant (Fig. 6d; Supplemental Fig. 10b, c). As expected, treatment with control CAR-M^GFP^ also inhibited melanoma growth compared to vehicle controls (Supplemental Fig. 10b, c), suggesting that adding any exogenous macrophages alone affects tumor growth as previously described [11, 14, 17]. Together these results suggest that CSPG4-targeting CAR-Ms may reduce melanoma growth in vivo.

We next modeled CAR-M treatment following tumor growth, and thus moved beyond same-day injection studies. We injected A375-H2B-mCherry cells into NRG mice, and first allowed the tumors to engraft for 5 days (Fig. 6e). We then peritumorally injected CAR-M^αCSPG4^ or controls (Fig. 6f) on day 5 and again on day 9. We again found no differences in animal weight across conditions (Supplemental Fig. 10d). Excitingly, we found that CAR-M^αCSPG4^-treated animals exhibited a significant reduction in tumor growth compared to CAR-M^GFP^-treated animals (Fig. 6g; Supplemental Fig. 10e, f), and that this inhibition of tumor growth began at day 22 and was maintained through day 27, well after CAR-Ms are no longer detected in the tumor. Immunofluorescence of the tumors at day 27 confirmed that the majority of CAR-Ms are no longer present in the tumor (Supplemental Fig. 10g), suggesting that the sustained inhibition of melanoma growth is likely mediated by reprogramming of the myeloid cells present in the tumor microenvironment. These results show that first, CSPG4-targeting CAR-Ms inhibit melanoma growth in vivo, and second, tumor growth inhibition occurs in the absence of adaptive immune cells, suggesting the innate immune system is sufficient for this response.

## Discussion

Current therapies for treating advanced stages of melanoma have improved patient survival, but primary resistance is still a relatively common occurrence, as well as recurrence via acquired resistance [62]. Pro-tumorigenic macrophages constitute a large portion of the tumor microenvironment in melanoma. Since CAR-T cells have been unsuccessful at infiltrating solid tumors, we hypothesized that CAR-Ms may effectively target melanoma tumors. In this study, we unveiled a potential treatment strategy for melanoma by CAR-Ms. Using CSPG4 as the target melanoma antigen, our findings demonstrate that CAR-Ms efficiently phagocytose metastatic melanoma cells. When CSPG4-targeting CAR-Ms are combined with CD47 blocking antibodies, thereby blocking a key tumor cell “don’t eat me” signal, we observe enhanced phagocytosis of melanoma cells and robust inhibition of melanoma spheroid growth in 3D. Importantly, we show that CSPG4-targeting CAR-Ms control tumor growth in vivo. We demonstrate that in a xenograft model, with mice lacking adaptive immune cells [61], CSPG4-targeting CAR-Ms exhibit sustained inhibition of melanoma growth. These data support the hypothesis that engineering macrophages with a melanoma-specific antigen is a promising therapeutic strategy for metastatic melanoma. Furthermore, our work provides the foundation for further work optimizing the CAR-M technology, the melanoma target, and the treatment regimen for melanoma.

Our findings raise important questions regarding how long CAR-Ms must persist at the tumor to maintain tumor control. If CAR-M-mediated phagocytosis of cancer cells is the primary mechanism for anti-tumor activity, then both sustained persistence and delivery of high CAR-M numbers are critical. Alternatively, if tumor immune reprogramming by CAR-Ms is the primary mechanism for anti-tumor activity, transient CAR-M persistence at the tumor may be sufficient for tumor control. Both mechanisms likely contribute to anti-tumor activity, and further work is required to parse out the contributions of each of these mechanisms. Our data show that CAR-Ms are not detectable in the melanoma tumor after approximately 5 days. This short time frame is consistent with other reports [11, 16]. Despite the short-term CAR-M persistence in the tumor, as in this study, CAR-Ms targeting multiple tumor antigens across a variety of models maintain tumor control [11, 13, 24]. These results and ours suggest that CAR-M-mediated phagocytosis of cancer cells is not sufficient to prevent tumor growth alone, and likely requires contributions from other stromal cells. We provide evidence that CSPG4-targeting CAR-Ms inhibit melanoma growth in immune-compromised animals over 18 days from the last CAR-M injection, well after CAR-Ms are no longer detected in the tumor [61]. These results suggest that innate immune cells are sufficient to inhibit melanoma growth in animals treated with CSPG4-targeting CAR-Ms. This inhibition is likely through local reprogramming of the tumor-associated macrophages into anti-tumor macrophages, thereby increasing phagocytosis of melanoma cells by endogenous macrophages, and/or by decreasing immunosuppressive signals at the tumor and promoting general anti-tumor responses. While our work suggests that innate immune cells can inhibit melanoma growth, CAR-Ms likely regulate the adaptive immune response, as has been previously suggested [11, 13, 22, 63]. Future CAR-M studies must perform systematic immune profiling at multiple time points over the growth of the tumor, dissecting each immune cell subset, their activation patterns, and exhaustion markers to provide a more in-depth understanding of the dynamics driving immune cell reprogramming in the tumor during CAR-M therapies. Furthermore, the number of CAR-Ms required, the injection frequency, as well as whether local versus systemic CAR-M injections provide better anti-tumor responses must be systematically tested and compared.

Teasing apart the requirement for CAR-M persistence at the tumor and CAR-M-mediated immune reprogramming will guide the development of next-generation CAR-Ms and combination therapies. We hypothesized that increasing CAR-M-mediated phagocytosis of cancer cells is a critical aspect of anti-tumor activity for three reasons: 1) increase cancer cell cytotoxicity directly through phagocytosis; 2) facilitate the repolarization of tumor-associated macrophages into anti-tumor macrophages; 3) increase antigen presentation to activate the adaptive immune arm. Thus, we focused our efforts on improving CAR-M-mediated phagocytosis of melanoma cells by engineering the CSPG4-targeting chimeric antigen receptor to contain the intracellular FcRγ phagocytic signaling domain and combining the CSPG4-targeting CAR-Ms with CD47 blocking antibodies. It remains to be seen whether this combination will prove effective for melanoma in vivo; however, other solid tumor models have demonstrated success with pairing αCD47 strategies with CAR-Ms both in vitro and in vivo [12, 24, 25, 64], although more work is required to determine the safest approach to inhibit CD47/SIRPα signaling in the clinic. Several approaches to inhibit CD47/SIRPα signaling are currently being tested in cancer, either specifically blocking CD47 or SIRPα with antibody-based or gene knockout approaches, as well as bispecific approaches that block both CD47 and SIRPα [65–68]. Macrophages deficient in SIRPα lead to enhanced anti-tumor effects [69]; however, how efficiently gene manipulations can be made in patient macrophages for therapy has not yet been shown. One advantage of antibody-based approaches over gene knockout approaches is that previous work has shown that blocking CD47 with an intact Fc portion promotes anti-tumor activity through antibody-dependent cellular phagocytosis [65, 70]. However, it should be noted that clinical trials using the human monoclonal CD47 blocking antibody used in this study, also known as Magrolimab, have been halted due to increased risk of death, highlighting the complexity behind strategies inhibiting CD47/SIRPα. Given the large number of compounds and approaches being tested to inhibit CD47/SIRPα, the safest and most efficacious approach remains to be determined. Future approaches to improve phagocytosis also include using activation domains more specific to the engulfment machinery such as the Rac2-E62K mutation [19] and targeting additional “don’t eat me” signals [71]. If sustained CAR-M persistence at the tumor is deemed to be critical for tumor growth control, then efforts to improve CAR-M survival such as overexpression of cytokines like M-CSF may be needed [72]. Another approach to improve CAR-M phagocytosis activity is to directly polarize CAR-Ms to a pro-inflammatory phenotype prior to administration [11]. This approach would have the benefit of immediately releasing cytokines to dampen the immunosuppressive environment of the tumor; however, it remains unknown whether this approach increases the potential for dangerous side-effects such as cytokine release syndrome [11]. Our work provides compelling evidence that CAR-Ms can be successfully engineered to reduce primary melanoma growth across in vitro and in vivo melanoma models. The next critical steps involve determining whether local administration of CAR-Ms at the time of melanoma tumor resection prevents local melanoma recurrence. Furthermore, future work is required to determine whether systemic versus local administration of CAR-Ms prevents melanoma metastases.

## Materials and methods

### Cell culture

A375 (RRID:CVCL_0132), YUMM1.7 (RRID:CVCL_JK16), B16F10 (RRID:CVCL_0159), cell lines were obtained from the ATCC. Short-tandem repeat confirmed 624-mel (RRID:CVCL_8054) and WM793 (RRID:CVCL_8787) cell lines were obtained from the Judson-Torres lab at the University of Utah. YUMM1.1 (RRID:CVCL_JK10), YUMM3.2 (RRID:CVCL_JK35), and YUMM5.2 (RRID:CVCL_JK43) cells were generously provided by Matthew Williams’ lab at the University of Utah. For detailed information about the generation of fluorescent cell lines, see attached supplemental methods file.

### Human primary cell isolation and CAR generation

Anonymous donor healthy human blood in either leukoreduction filters or apheresis cones were obtained from Associated Regional and University Pathologists, Inc (ARUP). Leukocytes were recovered from blood, CD14+ monocyte isolated by adhesion, and transduced as previously described [49].

### Phagocytosis flow assay

Macrophages and target cells cultured in monoculture were pooled for a 0-hour coculture. Phagocytosis percentages were determined by gating live Cells, singlets gates, CD11B+ macrophages, GFP+ for transduced macrophages, and setting an Lck-mScarlet+ / GFP+ gate for phagocytosis based on 0-hour coculture (cells pooled together just prior to flow). See Supplemental Fig. 2d for full gating strategy and supplemental materials and methods for additional information.

### 3D Spheroid growth assay

2500 A375-H2B-mCherry cells or YUMM1.7-H2B-mCherry cells were plated in biofloat plates for 72 h to generate spheroids. 15 K CAR-Ms or control CAR-Ms were then added to the wells along with 10 µg/mL of IgG control antibody or αCD47 for 10 days. Media was changed every 3 days by removing 100 µL of media and replacing it with fresh media and treatments. Spheroids were imaged every 8 h using a 10x/0.3 objective with an Incucyte SX5 analysis system, and H2B-mCherry total object integrated intensity and largest object area were quantified using the spheroid analysis module following spectral unmixing using Incucyte software (version 2020 C Rev1).

### Xenograft experiments

Animal experiments were approved by the Institutional Animal Care and Use Committee (IACUC) at the University of Utah and conducted with assistance from the Preclinical Research Shared Resource at Huntsman Cancer Institute Research. 250 K A375-H2B-mCherry cells were injected with Matrigel subcutaneously into 6–8 week-old female NOD.Cg-*Rag1*^*tm1Mom*^*Il2rg*^*tm1Wjl*^/SzJ (NRG) mice (RRID:IMSR_JAX:007799). Animals were then randomized across conditions based on tumor size prior to treatments. The investigator was blinded to group allocation as these experiments were performed by a core facility. The subsequent injections were performed peri-tumorally with vehicle and CAR-Ms only in PBS on days indicated. For same-day injections only, at first injection, vehicle or CAR-Ms were injected with the tumor cells in Matrigel. Tumor volume measurements were taken with calipers as indicated on graphs until experimental endpoints.

### Image analysis

All images were acquired with a Zeiss LSM 880 microscope or an Incucyte SX5 system. For images acquired with the LSM 880, selected z-planes were used to generate maximum intensity projections using Zen software. Linear adjustments to brightness and contrast were done with FIJI. Representative images were cropped and assembled with Adobe Photoshop (version 24.4.1) and Illustrator (version 27.5).

### Statistical analysis

All statistical analysis was done using GraphPad Prism and presented as mean values +/− standard error of the mean (SEM). Outliers greater than two standard deviations were removed from analysis. The figure legend indicates the statistical test used and the number of biological and technical replicates. To determine the number of biological replicates, we sought to detect a 50% difference in phagocytosis between conditions, assuming a 10% standard deviation. Thus, we required a minimum of 3 biological replicates for our studies. All technical replicates within each biological replicate are plotted as superplots, therefore, variation is shown on each graph. Two-tailed statistical tests were performed. Flow cytometry data were analyzed using FlowJo software (version 10.10.0). Because animal experiments were not performed across both male and female mice, we are not addressing sex as a biological variable in these studies.

All other methods are available under supplemental materials file.

## Supplementary information

CSPG4-targeting CAR-Ms fully engulf melanoma cell fragments in 3D.

CSPG4-targeting CAR-Ms trogocytose melanoma cells in 2D.

CAR-M αCSPG4 infiltrate A375-Lck-mScarlet spheroids.

CSPG4-targeting CAR-Ms exhibit increased infiltration of melanoma spheroids compared to control CAR-Ms.

Supplemental Figures

Supplementary Material

## Data Availability

The data generated in this study are available within the article and its corresponding supplementary data files. Expression profile data analyzed in this study were obtained from Gene Expression Omnibus (GEO) at GSE151091 [38], the Broad Single Cell portal [36] (GSE115978), and the human protein atlas [73].
