# Supplementary material for: Human CSPG4-targeting CAR-macrophages inhibit melanoma growth
Source: Oncogene. 2025 Mar 13;44(22):1665–77. doi: 10.1038/s41388-025-03332-0 (PMC12122381; doi:10.1038/s41388-025-03332-0)
Supplement: Supplementary file 5 — Supplemental Figures [file 41388_2025_3332_MOESM5_ESM.docx]

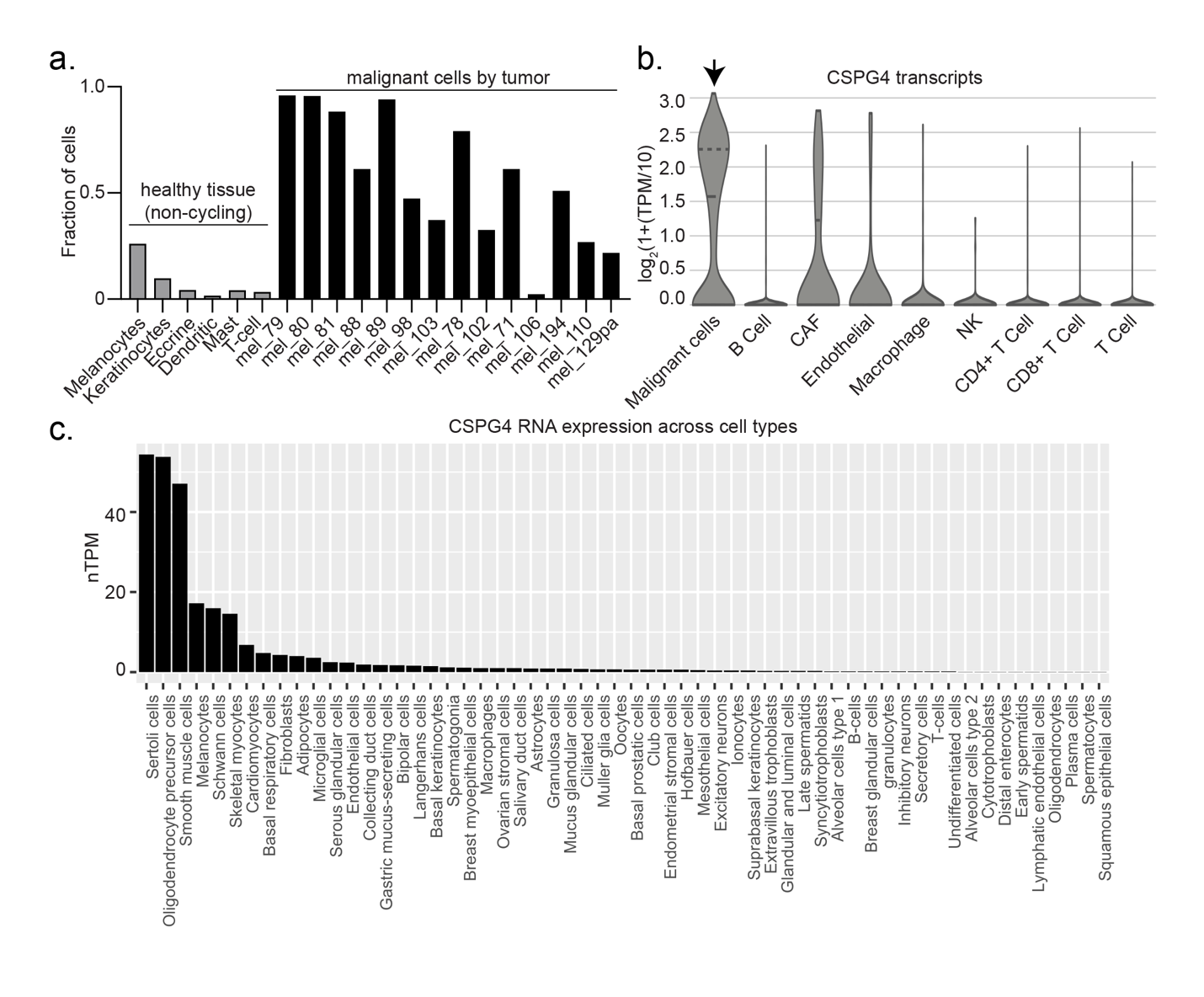


**Supplemental Figure 1|** CSPG4 expression is overexpressed and specific to melanoma. **a)** Fraction of cells expressing CSPG4 in single-cell RNA sequencing analysis of healthy (non-cycling, gray) tissue vs melanoma tumors (black) rank mean normalized from Figure 1A. **b)** Violin plot of CSPG4 transcript levels by single-cell RNA sequencing analysis of malignant cells (arrow) in tumors compared to non-malignant cells within the tumor. Quartiles are demarcated on the violin plots. **c)** CSPG4 transcript counts in normal cell types (Human Protein Atlas).

**
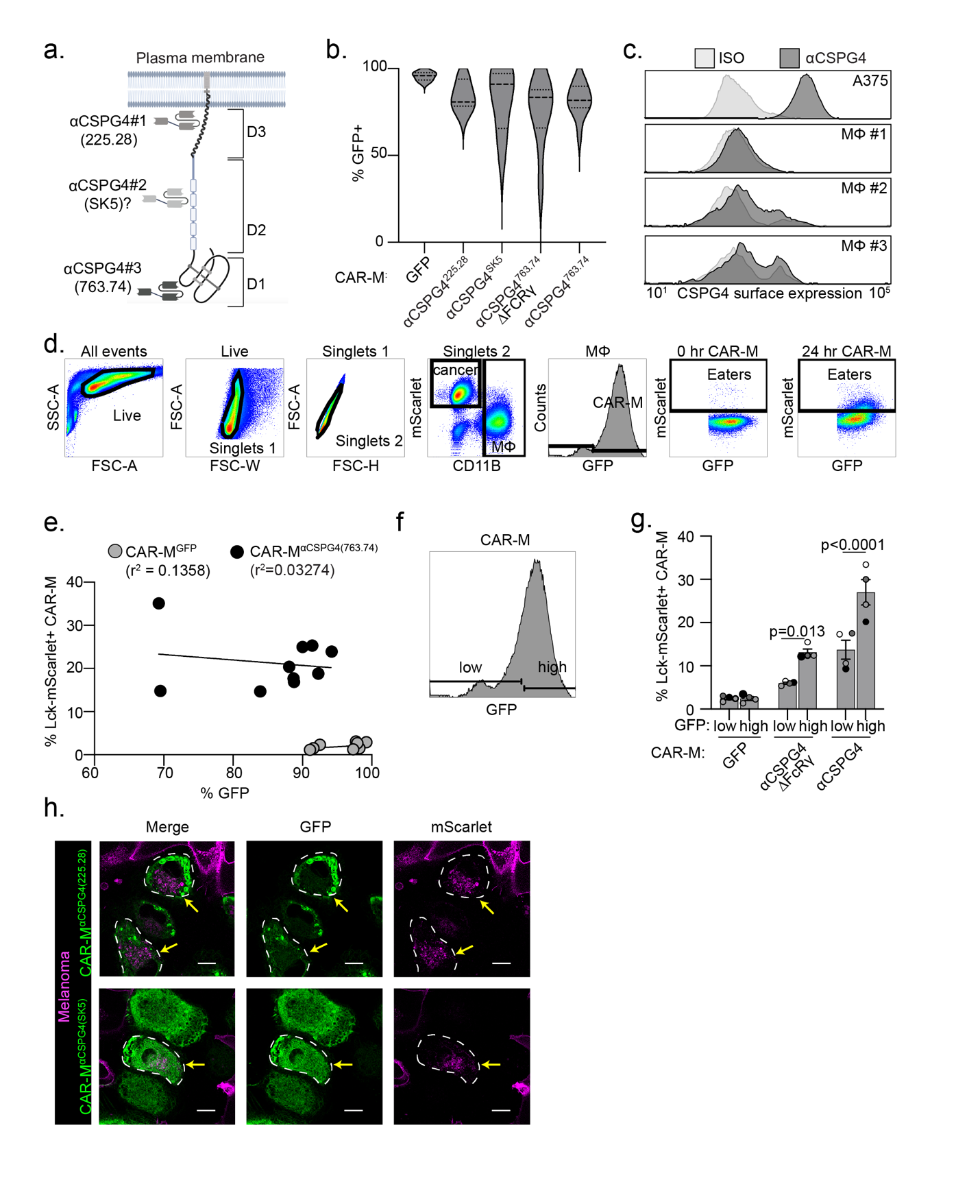
Supplemental Figure 2|** Engulfment gating strategy and CAR-M transduction efficiencies. **a)** Schematic (created with Biorender.com) of CSPG4 scFvs showing predicted (SK5) and validated (225.28, 763.74) domain binding. **b)** Violin plot of percentage of primary macrophages expressing each CAR-M construct (N: GFP = 12, 763.74 = 12, SK5 = 4, 225.28 = 3). **c)** CSPG4 surface expression by flow cytometry of A375 cells compared to primary macrophages (n=3 PBMC donors denoted as MΦ #1-3). **d)** Gating strategy for quantifying CAR-M transduction and CAR-M-mediated engulfment of A375-Lck-mScarlet cells based on 0-hour coculture. **e)** Comparison of CAR-M^αCSPG4(763.74)^ and CAR-M^GFP^ engulfment of Lck-mScarlet fragments and CAR expression from flow cytometry experiments with line of best fit (slope - CAR-M^GFP^: 0.086 and CAR-M^αCSPG4^: -0.13) and correlation. **f)** Gating strategy for segregating high vs low CAR expression on GFP control and CSPG4-targeting CAR-Ms. **g)** Quantification of CAR-M-mediated engulfment of A375-Lck-mScarlet cells by CAR-Ms with low or high CAR expression. **h)** Representative single z-plane images of CAR-M^αCSPG4(SK5)^ or CAR-M^αCSPG4(225.28)^ (green) and A375-Lck-mScarlet cells (magenta). The white dashed line outlines CAR-M^αCSPG4^ cell boundary, and the yellow arrows highlight engulfment events. Images acquired with a 63X objective on an LSM 880 microscope. Scale bar is 10 microns. g) Mean +/- SEM, 2-way AVOVA with Sidak’s multiple comparisons test. Non-significant comparisons are not indicated on the graphs.


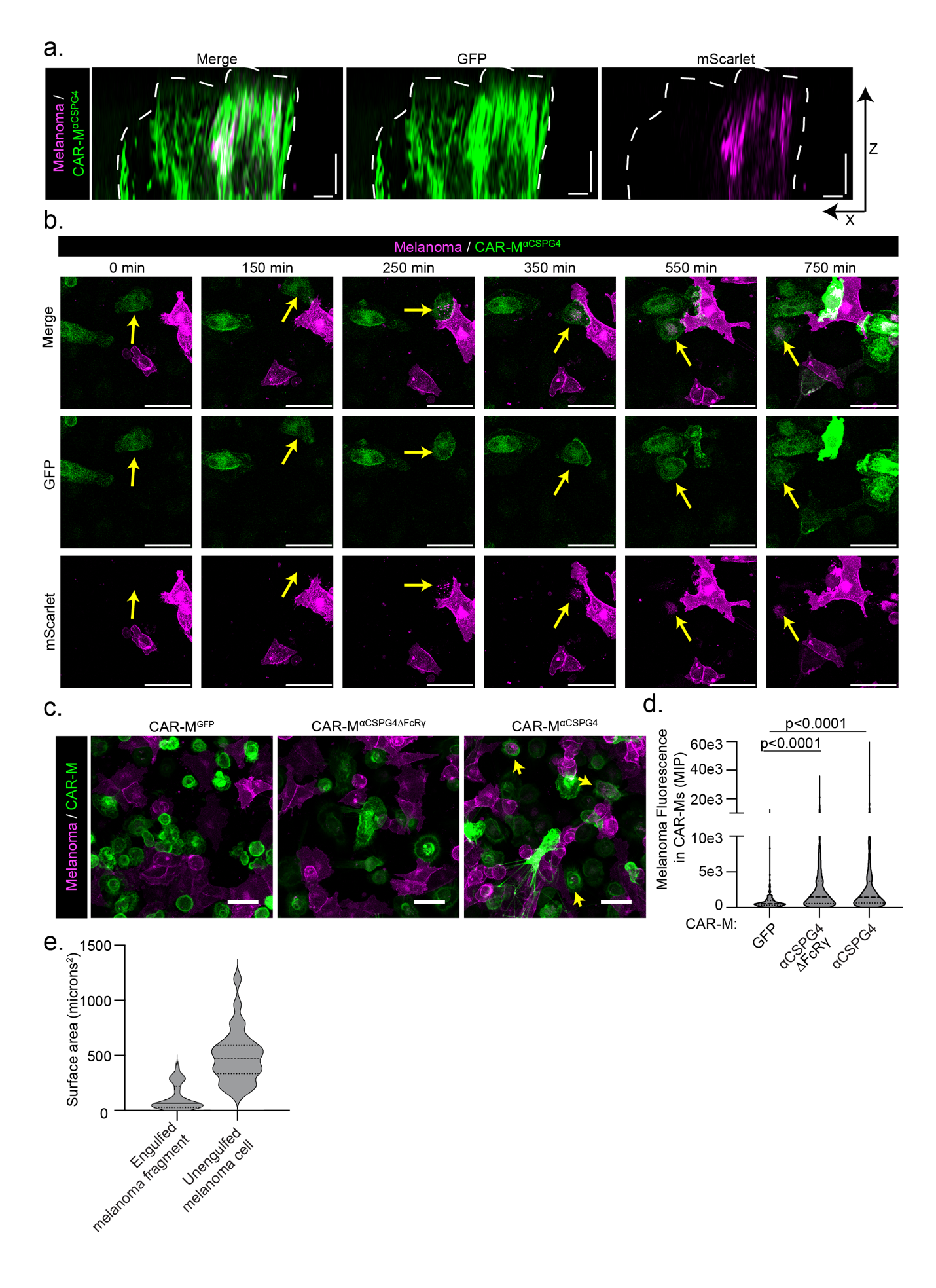

**Supplemental Figure 3|** Melanoma particles are fully internalized within CSPG4-targeting CAR-Ms. **a)** Representative X-Z plane images of engulfed Lck-mScarlet fragments inside of CAR-M^αCSPG4^ cells shown in Fig. 1g. Images acquired with a 63X objective on an LSM 880 microscope. Scale bars on both x and z axis represent 5 microns. **b)** Representative still images from timelapse recording (Supplemental Video 2) of CAR-M^αCSPG4^ (green) engulfment of Lck-mScarlet fragments (magenta). Yellow arrows highlight engulfment events. Scale bar is 50 microns. **c)** Representative images of A375-Lck-mScarlet cells cocultured with CAR-M^αCSPG4^ or CAR-M^GFP^ for 24 hours, images taken at 20X, yellow arrows indicate CAR-M eating events, scale bar is 40 microns. **d)** Violin plot, with quartiles, of quantification of Lck-mScarlet signal overlapping with GFP+ CAR-Ms in (c) from a maximum intensity projection (n=3 PBMC donors (biological replicates)). Mean +/- SEM, 1-way ANOVA with Tukey’s multiple comparisons test. **e)** Violin plot, with quartiles, of internalized Lck-mScarlet fragment area in CAR-M^αCSPG4^ (N= 34 engulfments, median = 64.69 microns^2^) or unengulfed A375-Lck-mScarlet cells (N = 28 cells, median = 473.19 microns^2^).

**
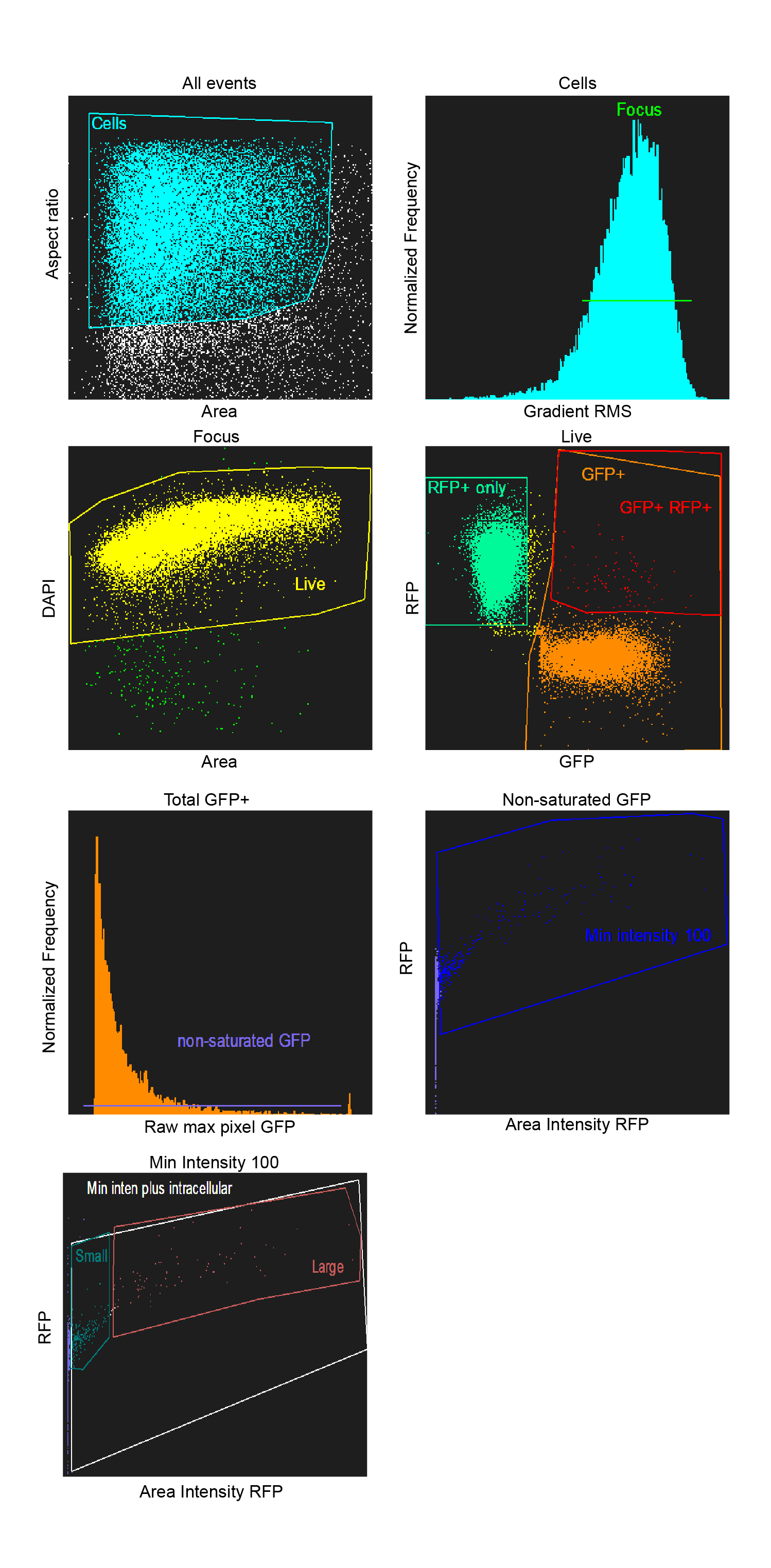
**

**Supplemental Figure 4|** Imagestream gating strategy for engulfment events. Imagestream gating strategy for identification of internalized Lck-mScarlet+ melanoma cell fragments in GFP+ CAR-M cells (data in Figure 3).

**
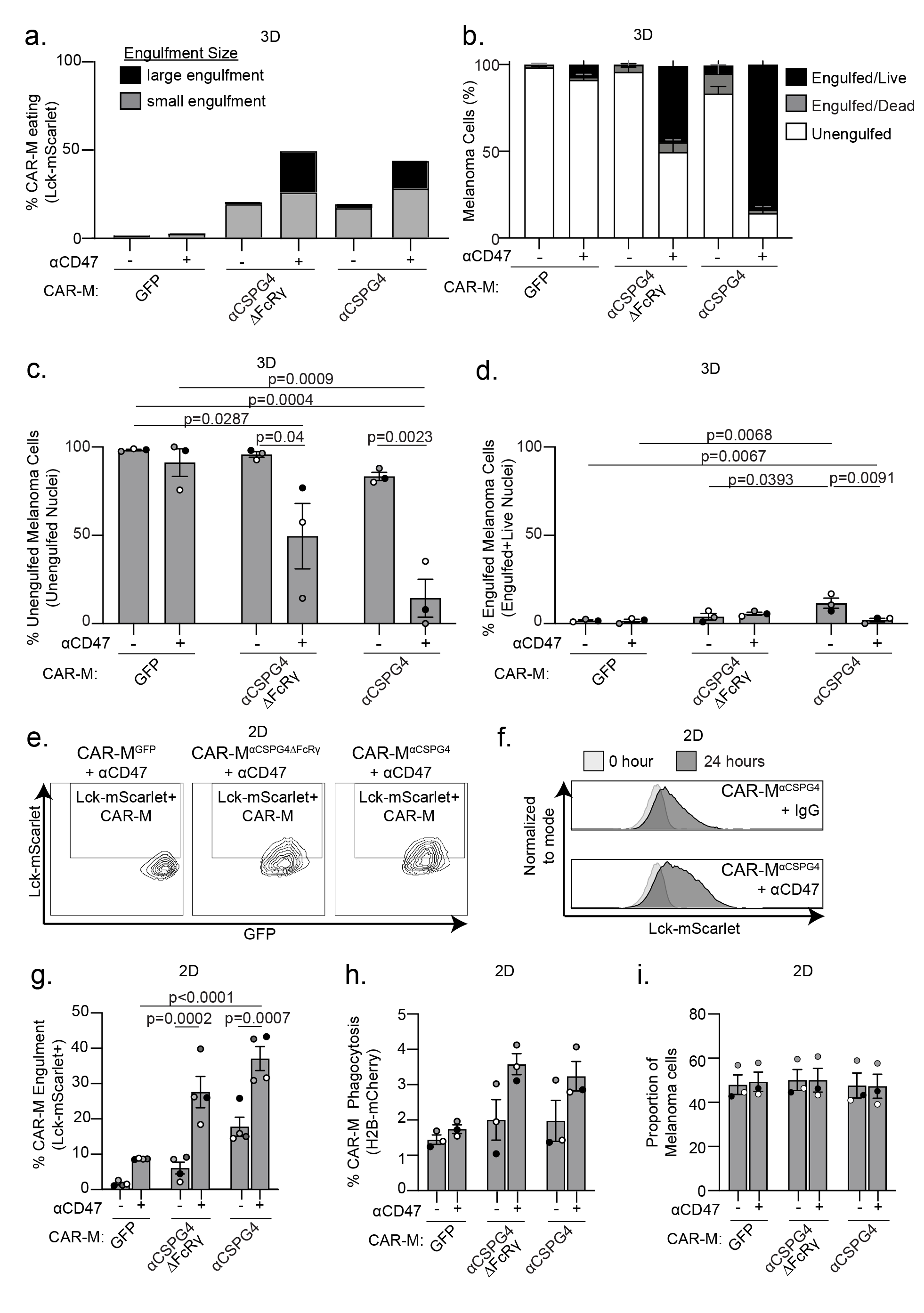
**

**Supplemental Figure 5|** αCD47 treatment increases CSPG4-CAR-M-mediated melanoma cell death in 3D, but not in 2D. **a)** Imagestream quantification of fully internalized A375 Lck-mScarlet punctae in GFP+ CAR-M cells upon treatment with 10 µg/mL αCD47 (+) or IgG isotype control (-) for 24 hours in 3D spheroids after 24 hours; images acquired with a 40X objective lens on an Imagestream. **b)** Classification of melanoma cells (live or dead) after 3 days of coculture with CAR-Ms as co-formed spheroids from Figure 4d. **c)** Quantification of unengulfed nuclei as percent of total melanoma cells after 72 hours of coculture in b. **d)** Quantification of engulfed nuclei as percent of total melanoma cells after 72 hours of coculture in b. **e)** Representative flow cytometry contour plots of CAR-M-mediated engulfment of melanoma fragments upon treatment with 10 µg/mL αCD47 (+) or IgG isotype control (-) for 24 hours in 2D culture. **f)** A normalized to mode histogram of Lck-mScarlet fluorescence in CAR-Ms at 0 and 24 hours in CAR-M^αCSPG4^ with 10 µg/mL αCD47 (+) or IgG isotype control (-) in 2D culture. **g)** Quantification of CAR-M-mediated engulfment of melanoma fragments upon treatment with αCD47 (+) or isotype control antibody (-) in (e, f). **h)** Quantification of CAR-M whole-cell phagocytosis of A375-H2B-mCherry cells treated with 10 µg/mL IgG isotype control (-) or αCD47 (+) in 2D culture. **i)** Proportion of target A375-H2B-mCherry cells remaining in coculture after 24 hours of coculture with CAR-Ms from (h). Each dot is a PBMC donor (biological replicate), as shades of gray. c, d, g, h, i) Mean +/- SEM, 2-way AVOVA with Tukey’s multiple comparisons test. Non-significant comparisons are not indicated on the graphs.

**
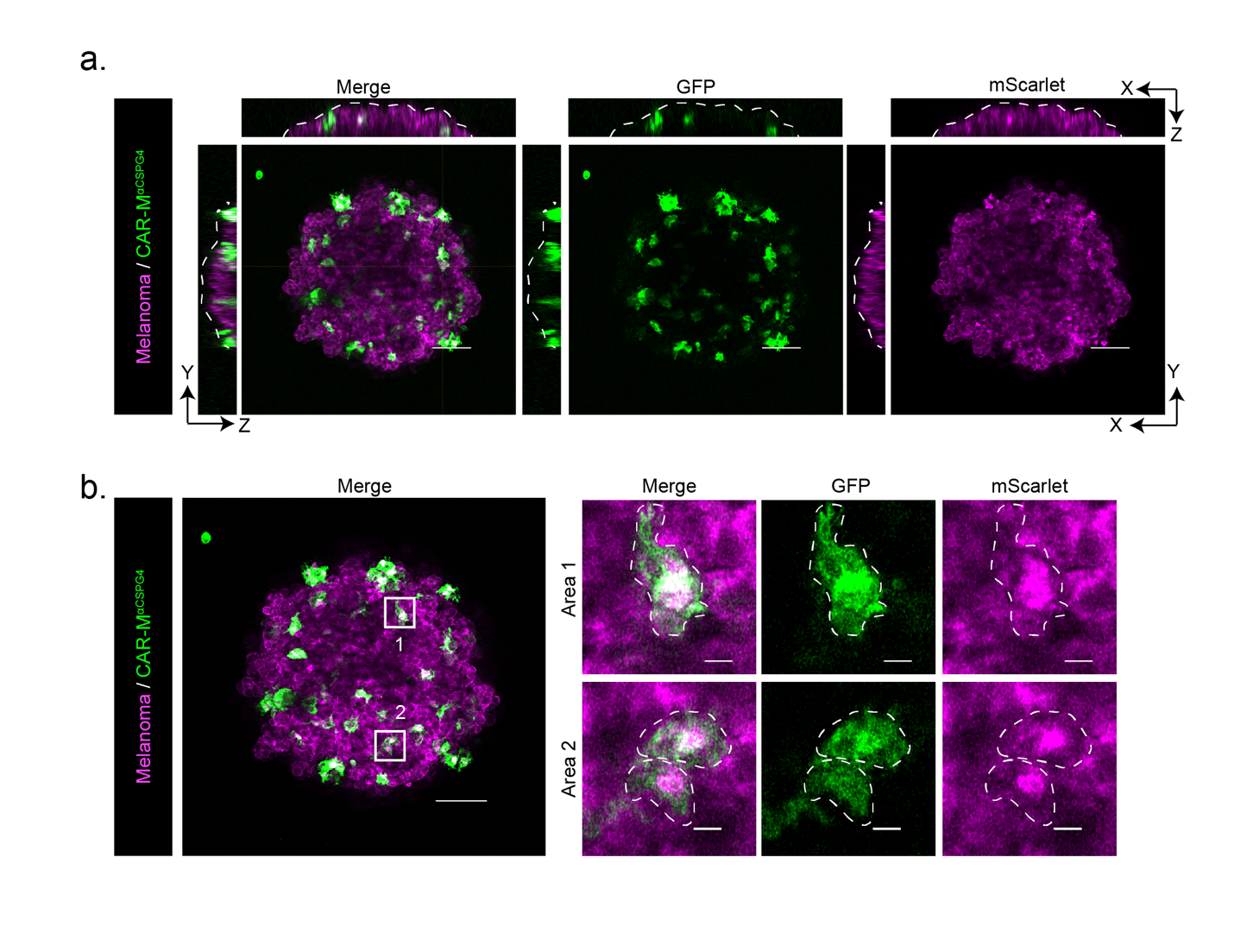
**

**Supplemental Figure 6|** CSPG4 CAR-Ms infiltrate melanoma spheroids and engulf melanoma cells. **a)** Representative still images with orthogonal x-z, and y-z slices of CAR-M^αCSPG4^ (green) inside A375-Lck-mScarlet spheroids (magenta) after 72 hours of coculture. Spheroid boundary is marked by white dashed line. Scale bar is 100 microns. Images acquired with a 10X objective. **b)** Representative maximum intensity projection of 3 slices indicating CAR-M (green) engulfment of mScarlet+ fragments. Two areas of the spheroid (boxes) are magnified to the right as Area 1 and Area 2, with CAR-Ms outlined in white hatched lines. Scale bar of main image is 100 microns; scale bar of inset is 10 microns.


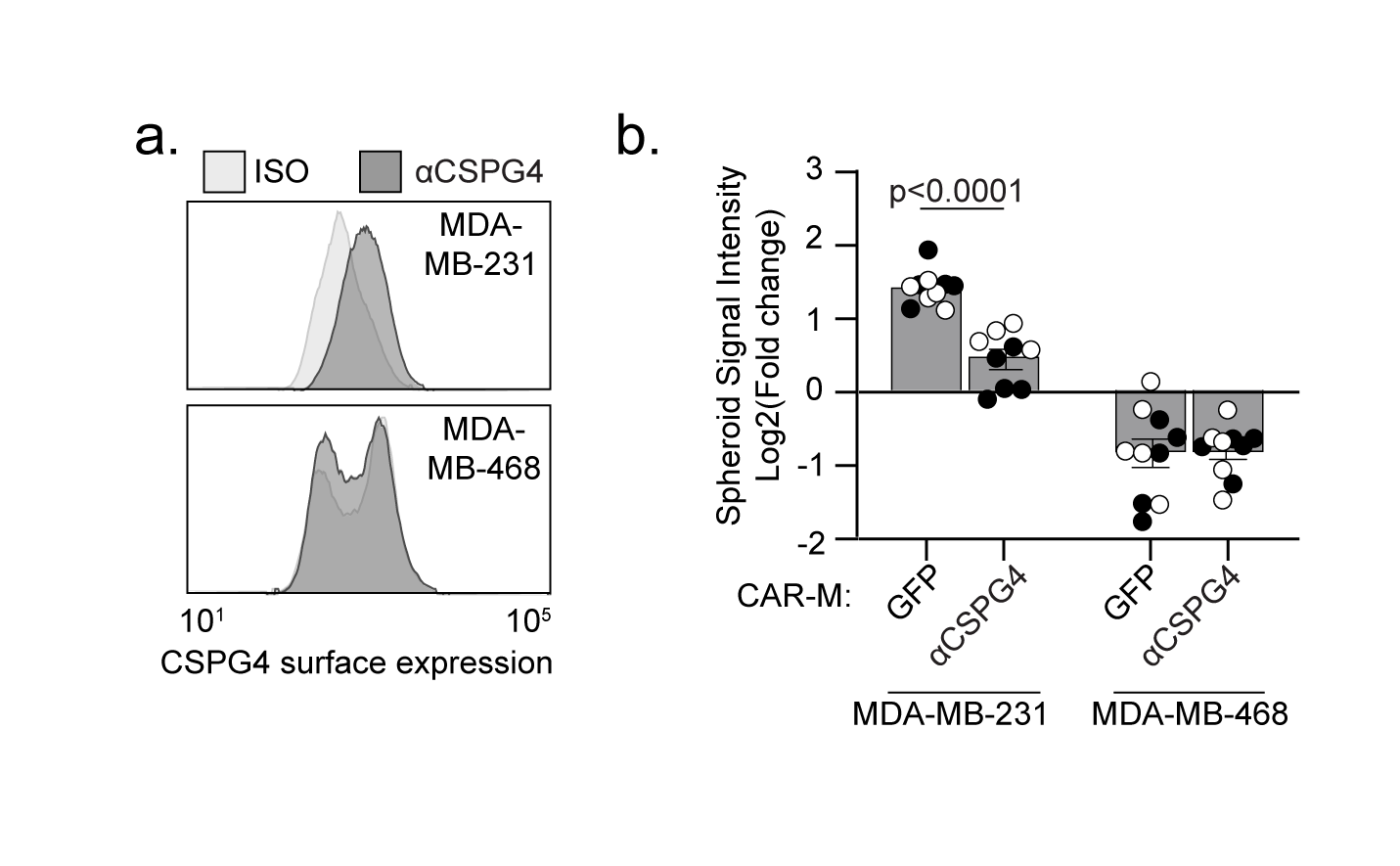


**Supplemental Figure 7|** CSPG4 CAR-Ms inhibit CSPG4+ breast cancer spheroid growth. **a)** Representative flow histograms of CSPG4 expression in MDA-MB-231 (CSPG4+) and MDA-MB-468 (CSPG4-) compared to isotype controls (ISO). **b)** Quantification of breast cancer spheroid growth as measured by log_2_ fold change in mCherry intensity after 12 days of coculture with CAR-M^GFP^ or CAR-M^αCSPG4^. Data shown as log_2_(fold change from time zero). Mean +/- SEM, 2-way ANOVA with Sidak’s multiple comparisons test. N=2 PBMC donors (biological replicates) as black or white dots, with each dot as a technical replicate. Non-significant comparisons are not indicated on the graphs.

**
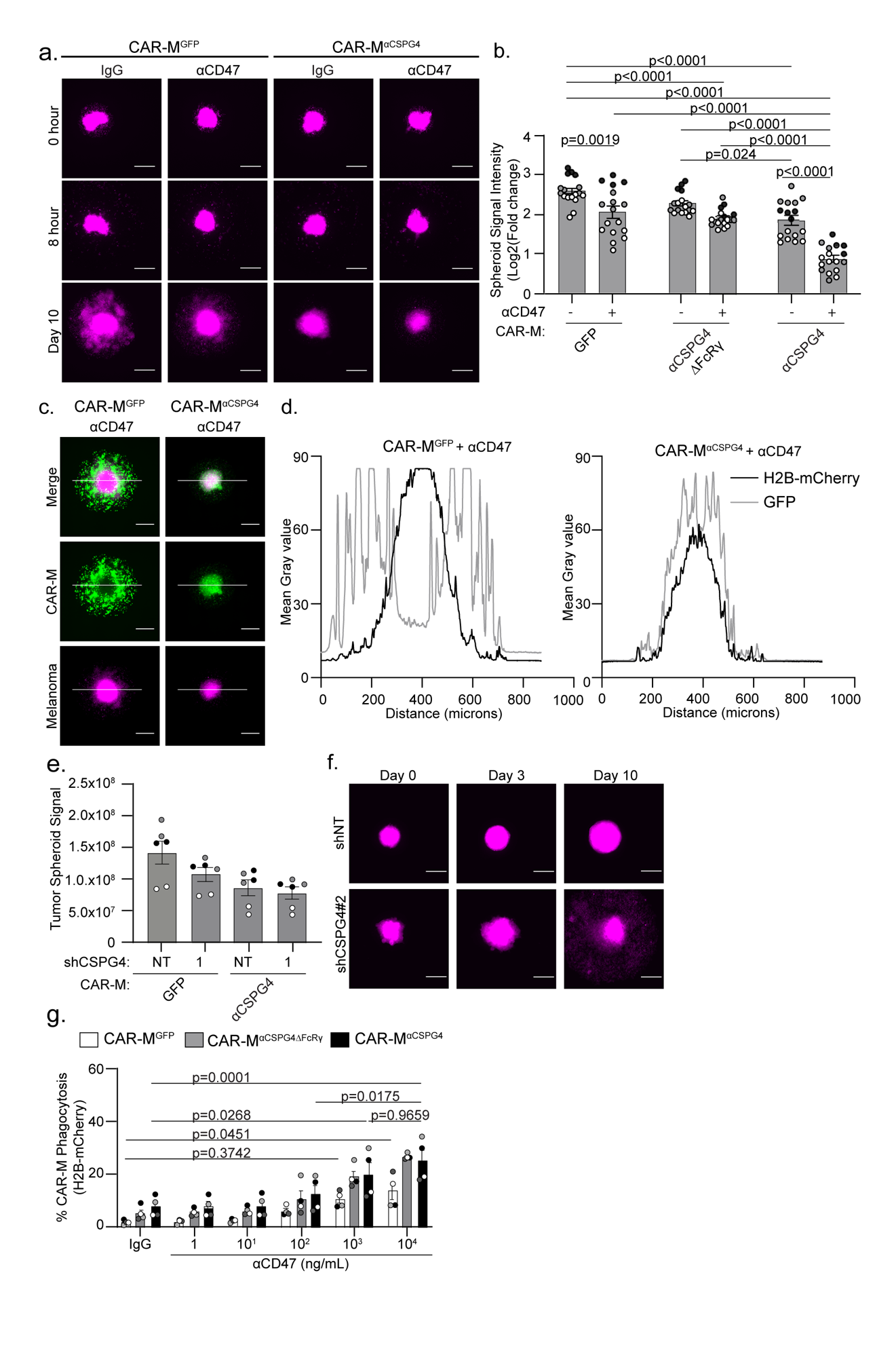
**

**Supplemental Figure 8|** Combining CSPG4-targeting CAR-Ms with αCD47 approaches leads to whole-cell melanoma phagocytosis in 3D. **a)** Representative images of pre-formed A375-H2B-mCherry spheroids (magenta) at day 0 (before CAR-Ms), 8 hours after CAR-M addition, and 10 days after CAR-M addition with 10 µg/mL αCD47 or IgG isotype control. Scale bar is 200 microns. **b)** Quantification of A375 spheroid growth as measured by total mCherry integrated intensity at day 10 across conditions (N = 17 spheroids across N=3 PBMC donors (biological replicates)). Data shown as log_2_(fold change from time zero). Mean +/- SEM, 2-way ANOVA with Tukey’s multiple comparisons test. **c)** Representative images of pre-formed A375-H2B-mCherry spheroids (magenta) and CAR-Ms (green) on day 10; white line indicates line scan measurements in (d). Scale bar is 200 microns. **d)** Line scan signal intensity from representative spheroid images in (c). **e)** Quantification of mScarlet integrated density of A375-Lck-mScarlet tumor spheroids after 10 days. A375-Lck-mScarlet cells were either treated with shNT or shCSPG4 RNA. **f)** Representative images of shNT and shCSPG4 #2 clones at day 0, day 3 (first media change), and day 10. Scale bar is 200 microns. **g)** Flow cytometry quantification of CAR-M-mediated whole-cell phagocytosis of A375-H2B-mCherry cells after 72 hours of 3D coculture with decreasing concentrations of αCD47 or IgG isotype control antibody. g) Mean +/- SEM, 2-way ANOVA with Tukey’s multiple comparisons test. N=4 PBMC donors (biological replicates). Non-significant comparisons are not indicated on the graphs, with the exception of on (g).

**
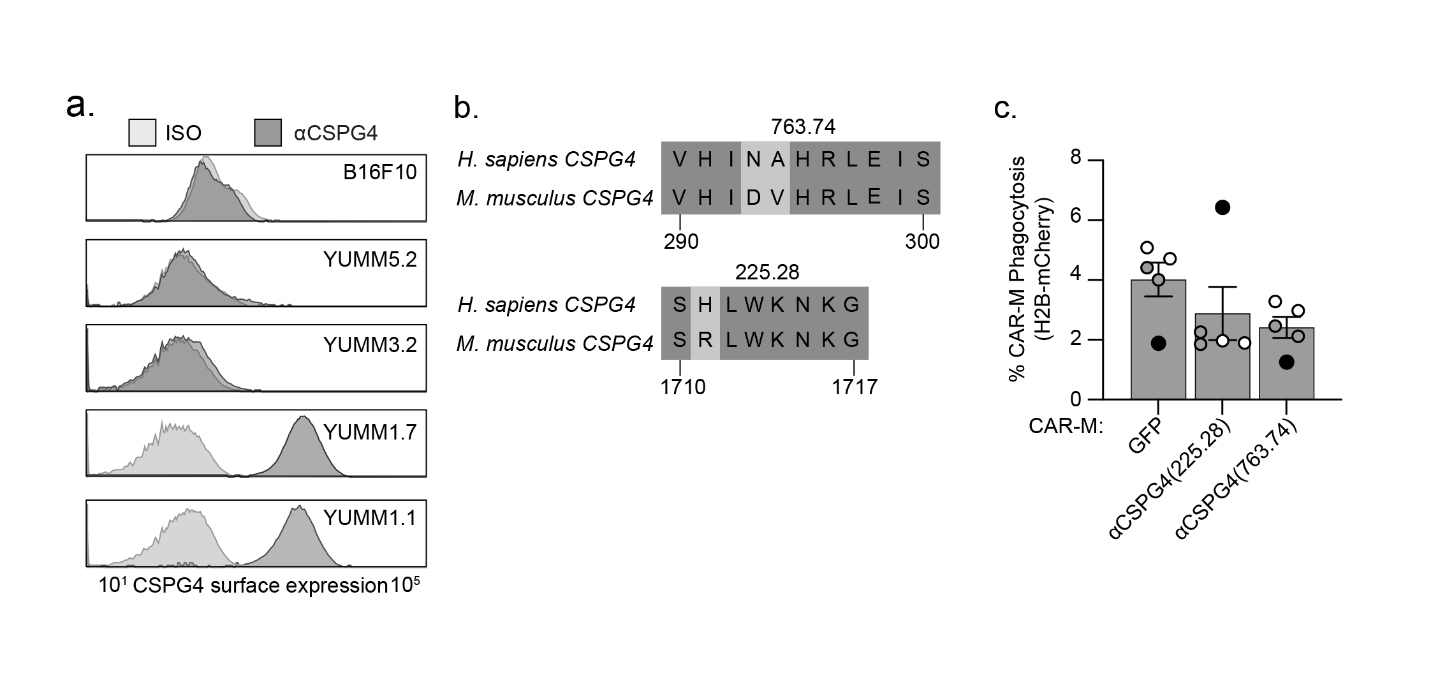
**

**Supplemental Figure 9|** CSPG4-targeting CAR-Ms using the 225.28 scFv do not efficiently phagocytose murine melanoma cells. **a)** Representative flow cytometry histograms of CSPG4 surface expression across a panel of murine melanoma cell lines. **b)** UniProt alignment of human vs mouse CSPG4 amino acid sequences for epitopes identified for 763.74 and 225.28 scFvs. **c)** Quantification of CSPG4^αCSPG4(225.28)^ whole-cell phagocytosis of YUMM1.7 H2B-mCherry murine melanoma cells compared to CAR-M^GFP^ and CAR-M^αCSPG4(763.74)^ by flow cytometry. N=3 PBMC donors (biological replicates) as shades of gray, and each dot as a technical replicate. 1-way ANOVA with Tukey’s multiple comparisons test, non-significant comparisons are not indicated on the graph.

**
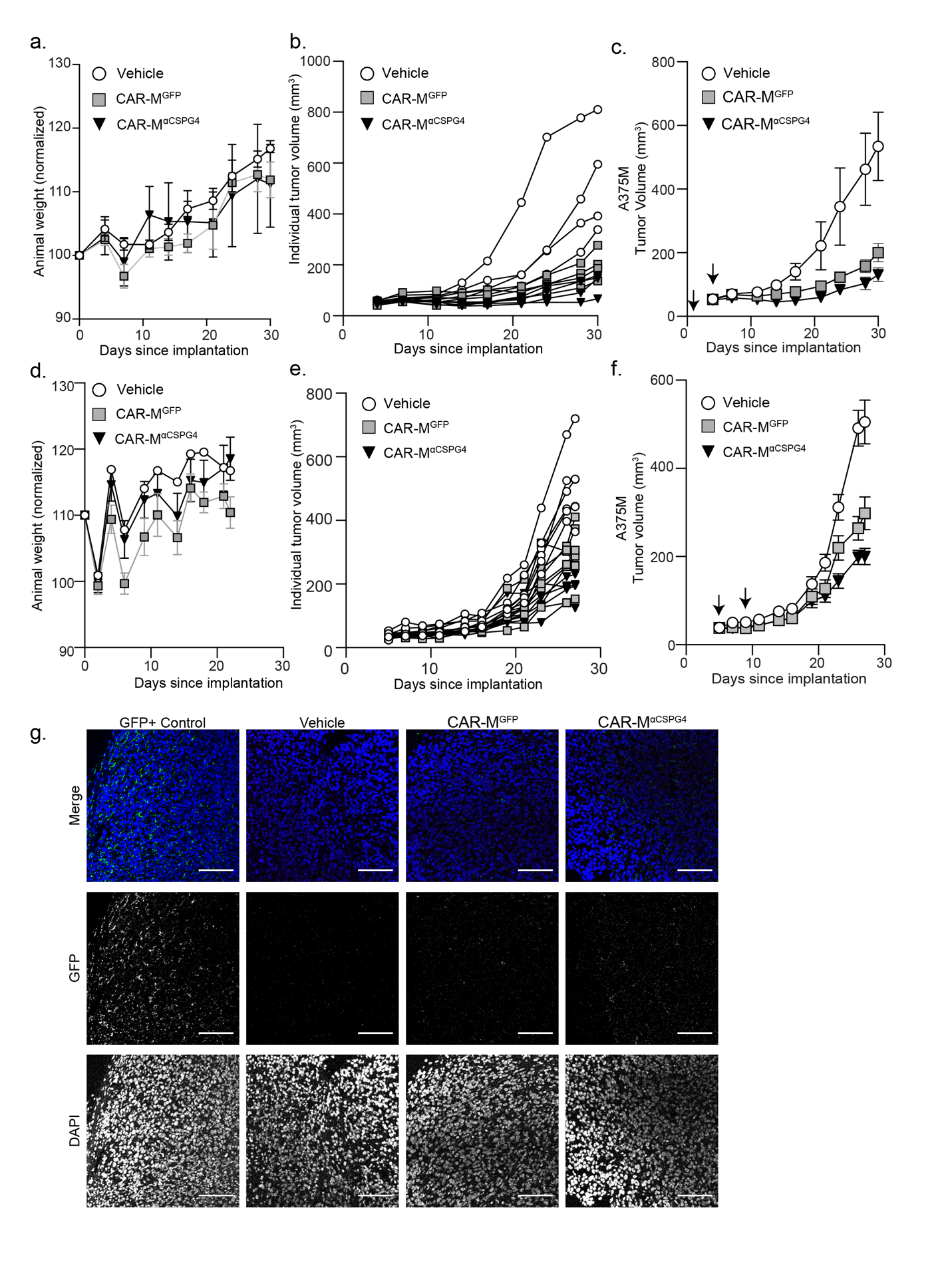
**

**Supplemental Figure 10|** CSPG4-targeting CAR-Ms do not affect mouse weight and do not persist in tumors 5 days after last peritumoral injection. **a-c)** Animal weights (a), individual mouse tumor volumes (b), grouped mouse tumor volumes including vehicle (c) from Figure 6d. **d-f)** Animal weights (d), individual mouse tumor volumes (e), and grouped mouse tumor volumes including vehicle (f) from Figure 6g. **g)** Representative 40X images of immunofluorescent staining for GFP and DAPI of tumors from Figure 6g. As a positive control for GFP staining, a tumor isolated from a GFP+ mouse was used. c, f) Mean +/- SEM. Arrows indicate CAR-M injections.

**Supplemental Video Legends**

**Supplemental Video 1|** CSPG4-targeting CAR-Ms fully engulf melanoma cell fragments in 3D. Video of sequential z-planes from confocal images of Fig. 1g – CAR-M^αCSPG4(763.74)^ (green) with internalized A375 cells tagged with Lck-mScarlet (magenta).

**Supplemental Video 2|** CSPG4-targeting CAR-Ms trogocytose melanoma cells in 2D. Timelapse video (maximum intensity projection) of CAR-M^αCSPG4(763.74)^ (green) interacting and trogocytosing/nibbling A375 cells tagged with Lck-mScarlet (magenta). Yellow arrows highlight CAR-M^αCSPG4(763.74)^ actively trogocytosing A375-Lck-mScarlet cell. Images taken every 10 minutes for 18 hours; 7 fps.

**Supplemental Video 3|** CAR-M ^αCSPG4^ infiltrate A375-Lck-mScarlet spheroids. Video of a Z-stack showing GFP+ CAR-M^αCSPG4^ (green) inside an A375-Lck-mScarlet spheroid (magenta).

**Supplemental Video 4|** CSPG4-targeting CAR-Ms exhibit increased infiltration of melanoma spheroids compared to control CAR-Ms. Timelapse video of CAR-M^GFP^ (green) that remain on the exterior of A375-H2B-mCherry spheroids (left side) compared to CAR-M^αCSPG4^ which infiltrate A375-H2B-mCherry spheroids (right side). Images taken every 8 hours for 10 days; 7 fps.
