# Supplementary material for: Human CSPG4-targeting CAR-macrophages inhibit melanoma growth
Source: Oncogene. 2025 Mar 13;44(22):1665–77. doi: 10.1038/s41388-025-03332-0 (PMC12122381; doi:10.1038/s41388-025-03332-0)
Supplement: Supplementary file 6 — Supplementary Material [file 41388_2025_3332_MOESM6_ESM.pdf]

Extended Supplemental Materials

Cell culture

A375 (RRID:CVCL_0132), YUMM1.7 (RRID:CVCL_JK16), B16F10 (RRID:CVCL_0159), MDA-MB-231 (RRID:CVCL_0062), MDA-MB-468 (RRID:CVCL_0419) cell lines were obtained from the ATCC. Short-tandem repeat confirmed 624-mel (RRID:CVCL_8054) and WM793 (RRID:CVCL_8787) cell lines were obtained from the Judson-Torres lab at the University of Utah. YUMM1.1 (RRID:CVCL_JK10), YUMM3.2 (RRID:CVCL_JK35), and YUMM5.2 (RRID:CVCL_JK43) cells were generously provided by Matthew Williams' lab at the University of Utah. A375 cells were cultured in DMEM (Thermofisher #11965118) supplemented with 10% FBS. 624-mel and WM793 cells were cultured in RPMI (Thermofisher #11875119) supplemented with 10% fetal bovine serum (FBS) (Sigma Aldrich #F4135) and 1% Glutamax (Thermofisher #35050061). YUMM1.1, YUMM1.7, YUMM3.2, and YUMM5.2 cells were cultured in DMEM/F12 (Thermofisher #11330057) supplemented with 10% FBS and 1% MEM non-essential amino acids (Thermofisher #11140050). Cell lines were routinely tested for mycoplasma detection using a PCR detection kit (ATCC, #30-1012K). Cells were used for <20 passages. Primary cells were cultured with RPMI supplemented with 10% FBS (Sigma Aldrich #F4135), 1% HEPES (Thermofisher #15630080), 1% Penicillin-Streptomycin (Sigma Aldrich #P4333), 0.1% 2-Mercaptoethanol (Thermofisher #21985023) and 20 ng/mL recombinant human GM-CSF (Peprotech #300-03).

Constructs

<u>Source plasmids:</u>

Lck-mScarlet-I (RRID:Addgene_98821)

pLKO-MCS (RRID: Addgene_185594)

pHR CD19-FcGamma CAR (RRID:Addgene_113014)

YFP-tagged FRB (RRID:Addgene_20148)

pHR CD22-empty -CAR (RRID:Addgene 113017)

pME-MCS_p2a_eGFP-CXXX (Gift from Dr. Cecilia B. Moens)

Anti-CSPG4 scFv SK5 (See below)

Anti-CSPG4 scFv 225.28s (See below)

SFG.scFv763.74.VK2VH1.hCD8a.CD28z (UNC IRB No. 18-0087)[46]

Plasmid construction

All plasmids were constructed via Gibson assembly. Fragments were amplified using NEB High Fidelity PCR Master Mix with HF Buffer (NEB #M0531S), followed by gel purification, and assembled using NEBuilder HiFi DNA Assembly Master Mix (NEB #E2621S).

FRB-CAR-M

FRB-CAR-M-GFP: Plasmid was constructed by amplifying fragments from pHR CD19-FcGamma-CAR (primers: (1,2), (5,6)) and YFP-tagged FRB (primers: (3,4)).

FRB-EMPTY-CAR-M-GFP: Plasmid was constructed by amplifying fragments from pHR CD22-empty-CAR (primers: (7-8), (5,11)) and FRB-CAR-M-GFP (primers: 9,10).

SK5-CAR-M

SK5-CAR-M-GFP: Plasmid was constructed by amplifying fragments from FRB-CAR-M-GFP (primers: (7,12), (11,15)) and a synthesized plasmid containing the Anti-CSPG4 scFv SK5 sequence (primers: 13,14).

SK5-EMPTY-CAR-M-GFP: Plasmid was constructed by amplifying fragments from CD22-Empty-CAR (primers: (7,16), (11,19)) and SK5-CAR-M-GFP (primers: 17,18).

SK5-CAR-M-eGFP-CXXX: Plasmid was constructed by amplifying fragments from SK5-CAR-M-GFP (primers: (20,21), (24,25)) and pME-MCS_p2a_eGFP-CXXX (primers: 22,23).

SK5- EMPTY-CAR-M-eGFP-CXXX: Plasmid was constructed by amplifying fragments from SK5-EMPTY-CAR-M-GFP (primers: (20,21), (24,25)) and pME-MCS_p2a_eGFP-CXXX (primers: 22,23).

225.28-CAR-M

225.28-CAR-M-GFP: Plasmid was constructed by amplifying fragments from FRB-CAR-M-GFP (primers: (1,26), (6,29)) and a synthesized plasmid containing the Anti-CSPG4 scFv 225.28s sequence (primers: 27,28).

225.28-Empty-CAR-M-GFP: Plasmid was generated by amplifying fragments from 225-CAR-M (primers: (20,21), (24,25)) and pME-MCS_p2a_eGFP-CXXX (primers: 22,23).

225.28-CAR-M-eGFP-CXXX: Plasmid was constructed by amplifying fragments derived from 225-CAR-M-GFP (primers: (20,21), (24,25)) and pME-MCS_p2a_eGFP-CXXX (primers: 22,23).

225.28-Empty-CAR-M-eGFP-CXXX: Plasmid was constructed by amplifying fragments derived from 225-CAR-M-GFP (primers: (20,30),(24,25)) and pME-MCS_p2a_eGFP-CXXX (primers: 23,31).

<u>763-CAR-M</u>

763.74-CAR-M-eGFP-CXXX: Plasmid was constructed by amplifying fragments from SK5-CAR-M-P2A-eGFP-CXXX (primers: (20,32), (33,34),(25,35)) and SFG.scFv763.74.VK2VH1.hCD8a.CD28z (primers: 27,36)

763.74-EMPTY-CAR-M-eGFP-CXXX: Plasmid was constructed by amplifying fragments from SK5-Empty-CAR-M-P2A-GFP-CXXX (primers: (20,32), (33,34),(25,35)) and SFG.scFv763.74.VK2VH1.hCD8a.CD28z (primers: 27,36)

<u>Other</u>

PLKO-Lck-mScarlet: Plasmid was constructed by amplifying fragments from Lck-mScarlet-I (primers: 37, 38) and pLKO-MCS (primers: (39,40), (41,42).

<u>Anti-CSPG4 scFv SK5:</u>

*VH:*GAGGTGCAGCTGGTGGAGTCTGGGGGAGGTGTGGTACGGCCTGGGGGGTCCCTGAG
ACTCTCCTGTGCAGCCTCTGGATTCACCTTTGATGATTATGGCATGAGCTGGGTCCGCCAA
GCTCCAGGGAAGGGGCTGGAGTGGGTCTCTGGTATTAATTGGAATGGTGGTAGCACAGGT
TATGCAGACTCTGTGAAGGGCCGATTCACCATCTCCAGAGACAACGCCAAGAACTCCCTGT
ATCTGCAAATGAACAGTCTGAGAGCCGAGGACACGGCCGTGTATTACTGTGCAAGGGGCG
TGCTGTCGCGTTATTTTGACTACTGGGGCCAAGGTACCCTGGTCACCGTCTCGAGT

Linker: GGTGGAGGCGGTTCAGGCGGAGGTGGCTCTGGCGGTGGCGGATCG

*VL:*GAAATTGAGCTCACACAGTCTCCAGCCACCCTGTCTTTGTCTCCAGGGGAAAGAGCCA
CCCTCTCCTGCAGGGCCAGTCAGAGTGTTAGCAGCTACTTAGCCTGGTACCAACAGAAACC
TGGCCAGGCTCCCAGGCTCCTCATCTATGATGCATCCAACAGGGCCACTGGCATCCCAGCC
AGGTTCAGTGGCAGTGGGTCTGGGACAGACTTCACTCTCACCATCAGCAGCCTAGAGCCT

GAAGATTTTGCAGTTTATTACTGTCAGCAGCGTAGCAACTGGCCTCCGGCTTTCGGCGGAG
GGACCAAGGTGGAGATCAAACGTGCGGCCGCA

Anti-CSPG4 scFv 225.28s

*VH:*CAAGTCAAACTGCAGCAGAGCGGTGGAGGCCTGGTGCAGCCTGGTGGCAGCATGAAG
CTGAGCTGCGTCGTGAGCGGCTTCACCTTCAGCAACTACTGGATGAACTGGGTCCGGCAG
AGCCCCGAGAAGGGCCTGGAATGGATCGCCGAGATCCGGCTGAAAAGCAACAACTTCGGC
CGGTACTACGCCGAGAGCGTGAAGGGCCGGTTCACCATCAGCCGGGACGACAGCAAGAG
CAGCGCCTACCTGCAGATGATCAACCTGCGGGCCGAGGACACCGGCATCTACTACTGCAC
CAGCTACGGCAACTACGTGGGCCACTACTTCGACCACTGGGGCCAGGGCACCACCGTGAC
TGTCAGCAGC

Linker: GGTGGCGGTGGCTCGGGCGGTGGTGGGTCGGGTGGCGGCGGATCT

*VL:*GACATCGAGCTGACCCAGAGCCCCAAGTTCATGAGCACCAGCGTGGGCGACAGAGTGT
CCGTGACCTGCAAGGCCAGCCAGAACGTGGACACCAACGTGGCCTGGTATCAGCAGAAGC
CCGGCCAGAGCCCTGAGCCTCTGCTGTTCAGCGCCAGCTACAGATACACCGGCGTGCCCG
ACAGATTCACAGGCAGCGGCTCCGGCACCGACTTCACCCTGACCATCAGCAACGTGCAGA
GCGAGGACCTGGCCGAGTACTTCTGCCAGCAGTACAACAGCTACCCCCTGACCTTCGGCG
GAGGCACCAAGCTGGAAATCAAG

Primer Sequence 5’ to 3’

Primer 1

ACCAGTCACAGAAAAGCATCTTACGGATGGCATGACAGTAAGAGAATTATGCAGTGC

Primer 2	GCCAGAGGATCGGCCTGGCGGCGTGGAG
Primer 3	CGCCAGGCCGATCCTCTGGCATGAGATGTG
Primer 4	GCGTCGTGGTTCTAGTCTTTGAGATTCGTCGG
Primer 5	AAAGACTAGAACCACGACGCCAGCGCCG
Primer 6	GATGCTTTTCTGTGACTGGTGAGTACTCAACCAAGTCATTCTGAGAATAG
Primer 7	GGTTGAGTACTCACCAGTCACAGAAAAGCATCTTACGGATG
Primer 8	GCCAGAGGATGAGGGATCAGCAGAAAGGCAGGGT
Primer 9	GCTGATCCCTCATCCTCTGGCATGAGATGTG
Primer 10	CGCTGGCGTCGTGGTTCTAGTCTTTGAGATTCGTCG
Primer 11	TTCTGTGACTGGTGAGTACTCAACCAAGTCATTCTGAGAATAGTGTATG
Primer 12	GACTCCACCAGCTGCACCTCCGGCCTGGCGGCGTGGAGCAG
Primer 13	TGCTCCACGCCGCCAGGCCGGAGGTGCAGCTGGTGGAGT
Primer 14	GCTGGCGTCGTGGTTCTAGTTGCGGCCGCACGTTTGATCTC
Primer 15	AGATCAAACGTGCGGCCGCAACTAGAACCACGACGCCAGCGCCGC

Primer 16	GACTCCACCAGCTGCACCTCCTGAGGGATCAGCAGAAAGGCAGGG
Primer 17	TTTCTGCTGATCCCTCAGGAGGTGCAGCTGGTGGA
Primer 18	CTGGCGTCGTGGTTCTAGTTGCGGCCGCAC
Primer 19	ATCAAACGTGCGGCCGCAACTAGAACCACGACGCCAGCGCC
Primer 20	AGTCAGGCAACTATGGATGAACGAAATAGACA
Primer 21	AAGTTAGTAGCTCCGCTTCCACTTCCGGATCCCTGGGGTGGTTTCTCAT
Primer 22	CACCCCAGGGATCCGGAAGTGGAAGCGGAGCTACTAACTTCAG
Primer 23	ACTCTAGAGTCGCGGCCGCTTCAGGAGAGCACACACTTGC
Primer 24	GCAAGTGTGTGCTCTCCTGAAGCGGCCGCGACTCT
Primer 25	CAGCGATCTGTCTATTTCGTTCATCCATAGTT
Primer 26	CTCTGCTGCAGTTTGACTTGCGGCCTGGCGGCGTGGAGC
Primer 27	TGCTCCACGCCGCCAGGCCGCAAGTCAAACTGCAGCAGAGC
Primer 28	GCTGGCGTCGTGGTTCTAGTCTTGATTTCCAGCTTGGTGCCTCC
Primer 29	GCACCAAGCTGGAAATCAAGACTAGAACCACGACGCCAGCGC
Primer 30	CGGGAATTCCGGACCGGTACTCTGCAGTAAAGGGTGATAACCAGTGACAGG
Primer 31	TTATCACCCTTTACTGCAGAGTACCGGTCCGGAATTCCC
Primer 32	TAAGGTTCCTTCACAAAGATCCGGGGC
Primer 33	TTATGAGTGGGCCCCGGATCTTT
Primer 34	CTCAGGCCGAATTCCATGGCCGGCCTGGCGGC
Primer 35	CAGTTTCCTCAACGCGTACCACCACGACGCCAGCG
Primer 36	GCGTCGTGGTGGTACGCGTTGA
Primer 37	GGGGATCCGGTTTAGTGAACCGTCAGATCCG
Primer 38	GTACAGATATCTTACTTGTACAGCTCGTCCATGCC
Primer 39	AGATAGGTGCCTCACTGATTAAGCA
Primer 40	ACTAAACCGGATCCCCCTGGGG
Primer 41	GCTGTACAAGTAAGATATCTGTACAAGTAACGCCCG
Primer 42	CAGTGAGGCACCTATCTCAGC

Lentiviral particle production

pCMV-VSV-G (RRID:Addgene_8454), psPAX2 (RRID:Addgene_12260), and transgene plasmids were transfected into HEK293FT (RRID:CVCL_6911) cells to generate lentivirus media as previously described[47] and concentrated using Lenti-X concentrator (Takara Bio #631232) according to the manufacturers protocol.

Generation of fluorescent tumor cell lines

Lentivirus containing pLKO-Lck-mScarlet or pLenti6-H2B-mCherry was generated from HEK293FT cells as described in 'Lentiviral particle production'. Unconcentrated lentivirus was added to melanoma cells at a 1:1 ratio with fresh media containing polybrene (10 µg/mL). After 72 hours, the cells were flow sorted for appropriate expression of either mScarlet or mCherry. To generate shCSPG4 cell lines, lentivirus containing plasmids from either non-target short hairpin RNA (Sigma #SHC0020) or shCSPG4 (gene target NM_001897) (Sigma #TRCN0000422139 or Sigma #TRCN0000437747), psPAX2, and pCMV-VSV-G were generated from HEK293FT cells as described in 'Lentiviral particle production'. All short hairpin RNA constructs were cloned into the pLKO.1 backbone. Melanoma cells expressing Lck-mScarlet were transduced using concentrated lentivirus containing the short hairpin RNA. Cells were selected and maintained for plasmid uptake with puromycin selection (2 µg/mL, Sigma Aldrich #P9620).

Mouse primary cell isolation and CAR generation

Bone marrow monocyte-derived macrophages (BMDMs) were isolated from C57BL/6 (RRID: IMSR_JAX:000664) mice femurs as previously described[65]. BMDMs were cultured in RPMI supplemented with 10% FBS, 1% Penicillin-Streptomycin, 1% Glutamax, 1% Sodium Pyruvate (Thermofisher #11360070), 0.1% 2-Mercaptoethanol and 50 ng/mL recombinant human M-CSF (Peprotech #300-25). BMDMs were transduced with concentrated lentivirus on day 3 with subsequent media changes every 2 days.

2D Phagocytosis flow assay (Extended)

Cells were detached using TrypLE Express (Thermofisher #12604021) and counted. 150K CAR-Ms were plated with 50K melanoma cells per well of a 12-well plate for 24 hours. Cells were detached from the plate using Trypsin-EDTA (Thermofisher #25200056), neutralized with complete media, and then pelleted and supernatant removed. From this point on, cells were kept at 4°C. Cells were then stained with BV711-αCD11B (Biolegend #301344, RRID:AB_2563792) in flow buffer (DPBS (Thermofisher #14190250), 2% FBS, and 5 μM EDTA (Corning #46-0343-CI)) for 30 minutes, then 1 mL of PBS was added to dilute the antibody and then pelleted again. Cells were resuspended in an appropriate volume of flow buffer. Cells were analyzed on a BD LSR Fortessa (5 lasers: UV, 405, 488, 561, 640). Data were then quantified on FlowJo (version 10.10.0).

2D Phagocytosis imaging assay

150K A375-Lck-mScarlet cells and 300K CAR-Ms were plated together as described in 2D Phagocytosis flow assay on glass bottom imaging dishes (WPI #FD35-100) and cultured for 24 hours. Images were acquired with either a Plan-Apochromat 20X/0.8 or 63X/1.4 oil DIC M27 objective on a Zeiss LSM 880 Airy Scan microscope using either the LSM acquisition mode or Airyscan FAST acquisition mode (and subject to deconvolution using Zen software (Carl Zeiss)) with 'auto' settings. Images at 20X were acquired with a 1-micron step size, and images at 63X were taken with a 0.19-micron step size. Maximum intensity projections of images or a central z-plane were analyzed in FIJI [66] (version 2.14.0) such that an automated mask was generated around GFP+ macrophages. Lck-mScarlet signal intensity was measured inside the GFP+ mask. Lck-mScarlet signal surface area was manually masked on maximum intensity projection images (Supplemental Fig. 3e). For images showing x-z, or y-z representations (Supplemental Fig. 3a, 6a), images were resliced in FIJI, and then scaled on the z-axis to improve visibility.

2D Timelapse overnight imaging

A375-Lck-mScarlet cells and CAR-Ms were plated together as described in '2D Phagocytosis imaging assay' on glass bottom imaging dishes and cultured for 1 hour to allow the cells to settle. Images were acquired using a Plan-Apochromat 20X/0.8 objective on a Zeiss LSM 880 using Airyscan FAST acquisition mode every 10 minutes for 18 hours and maintained at 37°C and at 5% CO_2 with an on-stage incubator.

3D Phagocytosis flow assay

15K CAR-Ms were plated in biofloat ultra-low adhesion plates (Sarstedt #83.3925.400) with 2500 Lck-mScarlet or H2B-mCherry transduced melanoma cells for 24 or 72 hours with 10 µg/mL of IgG control antibody or αCD47. Four spheroids were pooled for each technical replicate, then pelleted, and the supernatant removed. Cells were processed for flow cytometry as previously described in '2D Phagocytosis flow assay'.

Macrophage priming assay

1M CAR-Ms were plated in monoculture or with 200K A375-H2B-mCherry cells on 10cm tissue culture plates for 48 hours. The cells were then detached using TrypLE Express and the GFP+ CAR-Ms were flow sorted on a Propel labs Avalon and plated for downstream assays.

Nuclei status assay

Spheroids were prepared as described in '3D Phagocytosis flow assay'. Spheroids were then dissociated into single cells by pipetting and plated on glass bottom imaging dishes for 6 hours for cells to adhere. Cells were fixed in 4% paraformaldehyde and 2% sucrose in PBS for 10 minutes at 37°C. Cells were then permeabilized with 0.01% Triton-X100 in DPBS and stained with DAPI. Images were acquired with an LD C-Apochromat 40x/1.1 W Korr M27 objective on a Zeiss LSM 880 microscope using LSM acquisition mode with a 1.22-micron step size. H2B-mCherry cells were manually counted for either unengulfed (A375-H2B-mCherry cell not surrounded by GFP+ macrophages), engulfed and live (A375-H2B-mCherry surrounded by GFP+ macrophage

in X, Y, and Z plane, with H2B-mCherry colocalizing with DAPI), or engulfed and dead (A375-H2B-mCherry surrounded by GFP+ macrophage in X, Y, and Z plane, with H2B-mCherry signal dispersed and not colocalizing with DAPI).

CAR-M adherence assay

5K A375-Lck-mScarlet cells were plated as described in '3D spheroid growth assay'. 15K CAR-M were added to the wells along with 10 µg/mL of IgG control antibody or αCD47 for 8 hours. The spheroids were then physically removed from the well with a wide-bore pipette tip, gently mixed 1:1 with Matrigel, and then added to imaging dishes. Images were then acquired of the spheroids with an EC Plan-Neofluar 10x/0.30 M27 objective on a Zeiss LSM 880 microscope using Airyscan FAST acquisition mode with a 2.89-micron step size. Images were subject to deconvolution using Zen software with 'auto' settings and then processed in FIJI to generate a mask of the Lck-mScarlet+ spheroid. The number of GFP+ cells that were attached to the spheroid was then quantified.

Breast cancer 3D spheroid growth assay

MDA-MB-231-H2B-mCherry cells or MDA-MB-468-H2B-mCherry cells were plated in biofloat plates and centrifuged at 280G for 3 minutes, 24 hours later collagen was added to a final concentration of 3 µg/mL and centrifuged at 100G for 3 minutes and incubated for an additional 72 hours to generate spheroids. Spheroid growth was then measured as described in '3D Spheroid growth assay' for 12 days.

CAR-M infiltration assay

5K A375-Lck-mScarlet cells were plated as described in '3D spheroid growth assay'. 15K CAR-M were added to the wells for 8 hours. The spheroids were then transferred to Matrigel on imaging dishes as described in 'CAR-M adherence assay'. The cells were cultured for 96 hours and then images of the spheroids were acquired and processed as described in 'CAR-M adherence assay'.

A z-projection of the upper five z-slices was made and the number of GFP+ cells that were inside the spheroid mask were quantified.

CSPG4 expression flow assay

100K cells were plated in a 6-well tissue culture dish and incubated for 72 hours. Cells were then scraped into fresh media and then pelleted at 300G for 5 minutes. Primary macrophages were stained with Human TruStain FcX (Biolegend #422302) for 10 minutes at room temperature. Cells were stained with αCSPG4 antibodies (RRID: AB_10870987 (Fig. 2a), RRID:AB_2922401) (Supplemental Fig. 8a) or isotype control antibodies (RRID: AB_470111) (1:40) for 30 minutes on ice and protected from light. Cells were washed with PBS, pelleted at 300G for 5 minutes, and stained with secondary antibody (RRID: AB_2535719) (1:200) for 30 minutes on ice, protected from light. Cells were then washed with PBS, then resuspended in an appropriate volume of flow buffer and analyzed for fluorescence expression on the Fortessa. Cells were analyzed for live cells, singlets as described in '2D Phagocytosis Flow Assay' and then gated for CSPG4+ cells against isotype-stained control cells.

Image cytometry

Cells were plated as described above in 2D Phagocytosis flow assay (Fig. 3b, c) or 3D phagocytosis flow assay (Supplemental Fig. 5a). After 24 hours of coculture, cells were prepared for flow as previously described in '2D Phagocytosis flow assay'. Cells were stained with DAPI for viability gating. Imaging cytometry was performed on an Imagestream Mk II (Amnis). Cells were gated by Area/Aspect ratio, in-focus, DAPI for viability, total GFP+ events, non-saturated GFP+ events. Internalization events were detected by setting a minimum RFP+ intensity and applying a GFP signal adaptive erode to detect an RFP+ signal within a GFP+ signal. Internalized phagocytosis events were classified by size based on the area intensity of the RFP+ signal (~75-microns).

Tumor digestion for flow cytometry

Tumors were mechanically dissociated with scalpels, then digested with RPMI (+.032% Aqueous Collagenase D, +.008% DNAse) for 40 minutes at 37°C on a shaker, inverting every 10 minutes. Digested tumor was then strained over 70-micron filters, washed in PBS, and then cells were counted for flow cytometry.

Tumor immunostaining and imaging

Tumors were harvested and fixed in formalin prior to embedding in paraffin. Samples were deparaffinized and rehydrated using Citrisolv (VWR #89426-268), followed by rinses in sequential dilutions (100%, 95%, 80%, 70%) of ethanol, and then ddH_2O and PBS. Antigen retrieval was done in Tris-EDTA (pH 9) buffer overnight at 60°C, and then washed 2x with ddH_2O and PBS. Samples were blocked in 5% goat serum in TBS-T (0.05%) for 1 hour, followed by primary chicken anti-GFP (ABCAM #13970) (1:500) for 1 hour, all at room temperature. Samples were washed in TBS-T, and then stained for 1 hour with Goat Alexa fluor 488 anti-Chicken (1:500) (Jackson Immuno #103-545-155) at room temperature. Autofluorescence was then quenched using 0.1% Sudan Black in 70% ethanol for 10 minutes at room temperature, and then stained with DAPI prior to mounting and imaging. Images were acquired with a Zeiss LSM880 microscope with an LD C-Apochromat 40x/1.1 W Korr M27 objective using LSM acquisition mode. We used E0771 tumors grown in mice expressing GFP-tagged mitochondria in macrophages (mice carrying both of the following transgenes: B6.Cg-Gt(ROSA)26Sortm1(CAG-EGFP)Brsy/J (RRID:IMSR_JAX:032290) and a B6.129P2-Lyz2tm1(cre)Ifo/J (RRID:IMSR_JAX:004781)) to set a threshold for positive GFP expression.

Bioinformatic analysis

CSPG4 normalized expression values from fresh resected healthy human skin and metastatic melanoma specimens were obtained from previously published single-cell RNA-sequencing

(scRNAseq) data sets GSE151091 and the Single Cell portal (https://portals.broadinstitute.org/single_cell/study/melanoma-immunotherapy-resistance)[34, 36]. Both studies were analyzed using smartseq 2 based pipelines and then compared using rank mean normalization. Only non-cycling healthy human skin cells were included in the analysis. Data visualization and statistical analyses were performed in GraphPad Prism (version 9.4.1), python (version 3.7.4), and R (version 4.3.2).
